# Aggregation behavior of nanoparticles: Revisiting the phase diagram of colloids

**DOI:** 10.3389/fmolb.2022.986223

**Published:** 2022-09-19

**Authors:** Margherita Bini, Giorgia Brancolini, Valentina Tozzini

**Affiliations:** ^1^ Istituto Nanoscienze—CNR, Lab NEST SNS, Pisa, Italy; ^2^ Istituto Nanoscienze—CNR, Center S3, Modena, Italy

**Keywords:** bio-functionalized metal nanoparticles, colloids, classical molecular dynamics, low-resolution models, effective potentials, aggregation phase diagrams

## Abstract

Surface functionalization of metal nanoparticles (NPs), e.g., using peptides and proteins, has recently attracted a considerable attention in the field of design of therapeutics and diagnostics. The possibility of diverse functionalization allows them to selectively interact with proteins, while the metal core ensures solubility, making them tunable therapeutic agents against diseases due to mis-folding or aggregation. On the other hand, their action is limited by possible self-aggregation, which could be, however, prevented based on the full understanding of their phase diagram as a function of the environmental variables (temperature, ionic strength of the solution, concentration) and intrinsic characteristics (size, charge, amount, and type of functional groups). A common modeling strategy to study the phase behavior is to represent the NPs as spheres interacting via effective potentials implicitly accounting for the solvation effects. Their size put the NPs into the class of colloids, albeit with particularly complex interactions including both attractive and repulsive features, and a consequently complex phase diagram. In this work, we review the studies exploring the phases of these systems starting from those with only attractive or repulsive interactions, displaying a simpler disperse-clustered-aggregated transitions. The phase diagram is here interpreted focusing on the universal aspects, i.e., those dependent on the general feature of the potentials, and available data are organized in a parametric phase diagram. We then consider the potentials with competing attractive short range well and average-long-range repulsive tail, better representing the NPs. Through the proper combination of the attractive only and repulsive only potentials, we are able to interpret the appearance of novel phases, characterized by aggregates with different structural characteristics. We identify the essential parameters that stabilize the disperse phase potentially useful to optimize NP therapeutic activity and indicate how to tune the phase behavior by changing environmental conditions or the NP chemical–physical properties.

## 1 Introduction

The interest toward bio-functionalized metal nanoparticles (NPs) has grown recently for their potential applications in the nanotechnology fields (Chen et al., 2015), especially nano-medicine (Vlamidis and Voliani, 2018). In particular, the gold NPs (Alex and Tiwari, 2015) allow functionalization with biomolecules via a sulfur-mediated covalent bonding (Häkkinen, 2012). This results in different types of functionalization capable of selectively favoring the interaction with proteins or other specific components of the cell milieu, with potential therapeutic use (Marcinko et al., 2017). However, their calibration to this aim is indeed complex (Liao et al., 2012). The tendency of proteins to aggregate, depending on their internal state [e.g., misfolding, (Goldschmidt et al., 2010), (Aulić et al., 2014), (Spagnolli et al., 2021)] and on environmental factors [e.g., concentration, temperature, ionic strength of the solution (Peggion et al., 2017), (Carrillo-Parramon et al., 2016)], must be contrasted with appropriately affine NPs (Palmal et al., 2014). The NP–protein interaction also depends on the intrinsic properties of the particle (especially the type of functionalization, but also size and charge) and on the environmental factors. On the other hand, the NP–NP affinity must be tuned to prevent that they themselves aggregate, which would reduce their therapeutic efficiency. In order to use the optimal type and concentration of NPs, it is therefore of paramount importance to understand the phase behavior of the single components (NPs and proteins) and of their mixture as a function of the environmental variables (temperature, concentration, and ionic strength of the solution) and of the intrinsic variables (size, charge, and functionalization).

Computer simulations are a valuable tool to address this problem accounting for all the variables. In this context, because atomistic simulations may not suffice to reach the very large time and size scales into play, the recursion to coarse-grained (CG) or multiscale models (Palermo et al., 2020), (Tavanti and Tozzini, 2014) emerges naturally. Computationally cheap implicit solvent, single-residue-level-based [“minimalist” (Trovato and Tozzini, 2012)] models for proteins have been optimized during these years (Di Fenza et al., 2009), (Delfino et al., 2020), (Delfino et al., 2019), using parameterization strategies that typically combine bottom-up with top-down approaches, i.e., including data from atomistic simulations, as far as thermo-statistic data or large dataset (Maccari et al., 2013; Spampinato et al., 2014) of structural data from the experiment, possibly with the aid of evolutionary algorithms (Mereghetti et al., 2017; Leonarski et al., 2013). Low resolution models for functionalized NPs appeared more recently (Angioletti-Uberti, 2017; Brancolini and Tozzini, 2019a) and displayed a large variety of approaches. The presence of the gold core and surface functionalization naturally suggests a multi-scale representation (Brancolini and Tozzini, 2019b) by means of a central large sphere decorated with smaller spheres (Tavanti et al., 2015a; Tavanti et al., 2015b; Radic et al., 2015), capable of accounting both of the global size and charge of the NP and of the surface chemical properties. This allows treating NP–protein interaction (Brancolini et al., 2019) accounting for the large variety of possible functionalization types.

On the route of the extreme simplification of the system, however, an alternative strategy is possible, i.e. treating the NPs (and proteins) as single spheroidal objects (Vácha et al., 2014), also called the meso-scale (MS) representation used, e.g., in some simplified models for the cytoplasm (Trovato and Tozzini, 2014). At this level of resolution, the considered systems naturally fall into the category of colloids, characterized by definition by large size and spheroidal shape, whose states and phase transitions were widely studied. Clearly, the interaction potential of these MS-NPs is indeed complex and may display both attractive and repulsive features (Lopez and Lobaskin, 2015), separated by a barrier of variable location and height, as an effect of the electrostatic long-range repulsion possibly coupled to the hydrophobic short-range attraction. This results in an extremely complex phase diagram with the emergence of additional phases.

In this work, we analyze the large amount of studies already done on the phases of these systems following an historical perspective, which leads from the hard sphere system to the colloids with competitive interactions. We analyze the appearance of each new phase as the effect of the additional new features of the potential, which often emerge as an effect of the frustration from repulsion and attraction. This allows us to give indications on how to tune the competing parts of the potential to control the behavior of NPs when put in a binary mixture with proteins, in order to optimize their therapeutic power.

## 2 Effective potentials for colloids: An historical perspective

Colloids are particles capable of giving rise to colloidal suspensions (sometimes called “colloids” themselves). If seen from a phenomenological point of view, a suspension is different from a solution because the dispersed particles are substantially larger than the solvent molecules. A more general definition is simply based on the size of the particles, regardless of their nature: to be a colloid, the particles must have a size between 1 and 1000 nm (Israelachvii, 2017). With this definition, both proteins (especially the globular ones, which tend to be spherical and less structured) and NPs can be considered colloids. While at the macroscopic level, it is responsible for the typical opalescent aspect, at the microscopic level, the large size of particles brings fundamental differences in the effective interaction potentials with respect to common inter-molecular or inter-atomic potentials. Consequently, substantially different phase behaviors may arise with respect to simple mixtures of fluids.

Here, we will consider only models treating the solvent (usually water) implicitly. Therefore, particles interact via an effective potential including all the solvent effects, namely, the dielectric polarization, the ionic screening, and the hydrophobicity. The simplest cases analyzed in this section are defined by a size parameter σ, delimiting a repulsive short-range wall, and a tail, which is attractive for hydrophobic particles (usually neutral), and repulsive for hydrophilic particles (charged or polar). The attractive case might seem at a first sight similar to its atomistic equivalent, e.g., the Lennard-Jones (LJ) system, while the repulsive case resembles a classical plasma, except for the fact that the particles are not in vacuum but embedded in an implicit solvent. Simple LJ and Coulomb potentials are not suitable to describe colloids: additional parameters are needed to correctly represent the ratio between the size and the interaction range. These already bring novel and interesting behaviors in the phase diagram. It is worth, however, analyzing also the behavior LJ, together with the hard spheres (HS) (Cowen and Carpenter, 2020), as paradigmatic reference systems.

### 2.1 Phase diagram of the non-penetrable spheres

On the conceptual level, the HS is the simplest effective potential, characterized by a single parameter, the sphere diameter σ, and a contact potential, null for interparticle distance *r>σ*, infinite for *r<σ*. The potential does not have any energy-dependent parameter, which implies that the behavior of the system is completely entropy-driven. It is customary to describe single-component HS systems by means of the compressibility factor z=pV/NkT identically equal to 1 for classical non-interacting particles (perfect gas; for the parameter definitions and their main relationships, Table 1). For the HS, the correction to *z* depends only on the particle density *ρ = N/V* (or concentration) and is usually expressed through the dimensionless parameter “packing fraction” $\eta =\frac{\pi }{6}\rho \sigma^{3}$

$$\frac{p}{\rho k_{B}T}=z\left(\eta \right)=1+f\left(\eta \right)=1+\overset{\infty }{\underset{i=2}{\sum }}B_{i}{\left(\frac{\eta }{\eta_{c}}\right)}^{i}$$ (1)

being $\eta_{c}=\pi \sqrt{2}/6$ ∼0.740 the maximum possible value of *η* found in the closed packed face-centered cubic (FCC) or hexagonal close packed (HCP) crystal. The last term in (1) is the virial expansion, with *B*_*i*_ related to the virial coefficients *b*_*i*_ by $B_{i}=b_{i}\rho_{c}^{i-1}$ and *ρ*_*c*_ the closed packing density, satisfying $\rho_{c}\sigma^{3}=\sqrt{2}=1.414$ . Analogously, the phase diagram does not depend on *T* but only on the pressure (or density).

**TABLE 1:** A summary of the properties and fundamental relationships of the different types of repulsive and attractive potentials

Potential		Parameters and relationships	Phases
Hard spheres (HS)	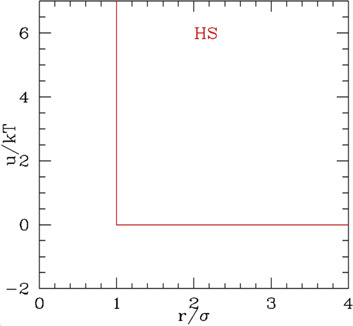	Diameter σ	Fluid
$u=\{\begin{array}{c} 0\;\;\;\;\;\;r\geq \sigma \;\\ \infty \;\;\;\;r<\sigma \end{array}\;$			
		Packing fraction $\eta =\frac{\pi }{6}\rho \sigma^{3}$	FCC
		Closed-packing $\eta_{c}=\frac{\pi \sqrt{2}}{6}$	glass
		Filling factor $\phi =\frac{\;\eta }{\eta_{c}}=\frac{\rho \sigma^{3}}{\sqrt{2}}$	
		Compressibility factor $z=\frac{p}{\rho kT}$	
Soft Spheres with inverse power law (IPL)	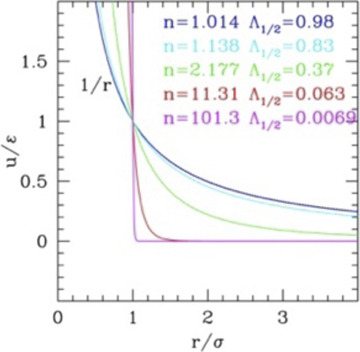	Diameter σ	Fluid
$u\left(r\right)=\varepsilon {\left(\frac{\sigma }{r}\right)}^{n}$			
		Range ∼σ (2^1/n^ -1)	FCC
		Force parameter ε	BCC
		Reduced temperature $\;\;\;\;\;\tau =\frac{kT}{\varepsilon }$	
		Scaling parameter $\gamma =\frac{6}{\pi }\eta \tau^{-\frac{3}{n}}=\frac{6}{\pi }\eta {\left(\frac{\varepsilon }{\;k_{B}T}\right)}^{\frac{3}{n}}$	
Yukawa with repulsive wall (HSY)	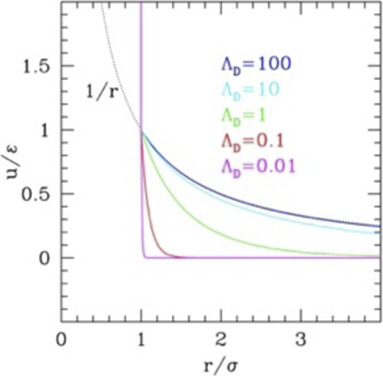	Screening (Debye) length $\;\lambda_{D}=\sqrt{\frac{\varepsilon_{0}k_{B}T}{8\pi e^{2}\left(n_{+}+n_{-}\right)}}$	Fluid
$u=\{\begin{array}{c} k_{B}T\lambda_{B}Q^{2}\frac{e^{-\frac{r}{\lambda_{D}}}}{r}\;\;\;\;\;r\geq \sigma \\ \infty \;\;\;\;\;\;\;\;\;\;\;\;\;\;\;\;\;\;\;\;\;\;\;\;\;\;\;\;\;\;\;\;r<\sigma \end{array}$			
$u=\{\begin{array}{c} \varepsilon \frac{e^{-\frac{r-\sigma }{\lambda_{D}}}}{r/\sigma }\;\;\;\;\;\;\;\;\;\;\;\;\;\;\;\;\;\;\;\;\;r\geq \sigma \\ \infty \;\;\;\;\;\;\;\;\;\;\;\;\;\;\;\;\;\;\;\;\;\;\;\;\;\;\;\;\;\;\;\;r<\sigma \end{array}$			FCC
		Bjerrum length $\lambda_{B}=\frac{e^{2}}{4\pi \varepsilon_{r}\varepsilon_{0}k_{B}T}$	BCC
		Effective charge $Q=Z\frac{e^{\frac{\sigma }{2\lambda_{D}}}}{\left(1+\frac{\sigma }{2\lambda_{D}}\right)}$	
		Reduced inverse temperature $\frac{1}{\tau }=\frac{\varepsilon }{kT}=\frac{\lambda_{B}{\left(1+\frac{\sigma }{2\lambda_{D}}\right)}^{2}}{\sigma Z^{2}e^{2}}$	
Pointlike Yukawa (PY)	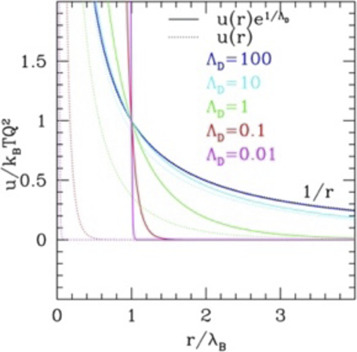	Accessible sphere radius $a={\left[\frac{3}{4\pi \rho }\right]}^{1/3}\;$	Fluid
$u\left(r\right)=\frac{Q^{2}}{4\pi \epsilon_{0}}\frac{{\text{e}}^{-r/\lambda_{D}}}{r}$			
		Scaling parameter $\text{Γ}=\frac{Q^{2}}{k_{B}T4\pi \epsilon_{0}a}=\frac{Q^{2}}{k_{B}T4\pi \epsilon_{0}}{\left[\frac{4\pi \rho }{3}\right]}^{\frac{1}{3}}$	BCC
Lennard-Jones (LJ)	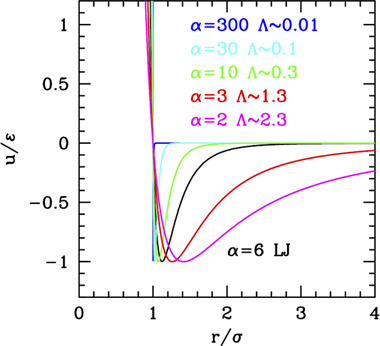	Diameter σ	gas
$u\;=4\varepsilon \;\left[{\left(\frac{\sigma }{r}\right)}^{12}-\;{\left(\frac{\sigma }{r}\right)}^{6}\right]$		Packing fraction $\eta =\frac{\pi }{6}\rho \sigma^{3}$	liquid
Generalized α-2α LJ		Attractive well ε	solid
$u{\;}^{\alpha }=4\varepsilon \;\left[{\left(\frac{\sigma }{r}\right)}^{2\alpha }-\;{\left(\frac{\sigma }{r}\right)}^{\alpha }\right]$		Reduced temperature $\;\;\;\;\;\tau =\frac{kT}{\varepsilon }$	
		Range λ∼3/α	
Hard-core attractive Yukawa (HAY)	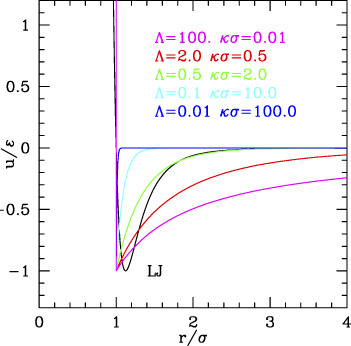	Diameter σ	gas
$u=\{\begin{array}{c} -\varepsilon \frac{e^{-\frac{r-\sigma }{\lambda }}}{r/\sigma }\;\;\;\;\;\;\;\;\;\;\;\;\;\;\;\;\;\;\;\;\;r\geq \sigma \\ \infty \;\;\;\;\;\;\;\;\;\;\;\;\;\;\;\;\;\;\;\;\;\;\;\;\;\;\;\;\;\;\;\;r<\sigma \end{array}$		Packing fraction $\eta =\frac{\pi }{6}\rho \sigma^{3}$	liquid
		Attractive well ε	solid
		Reduced temperature $\;\;\;\;\;\tau =\frac{kT}{\varepsilon }$	
		Range λ=1/κ	

**FIGURE 1:**
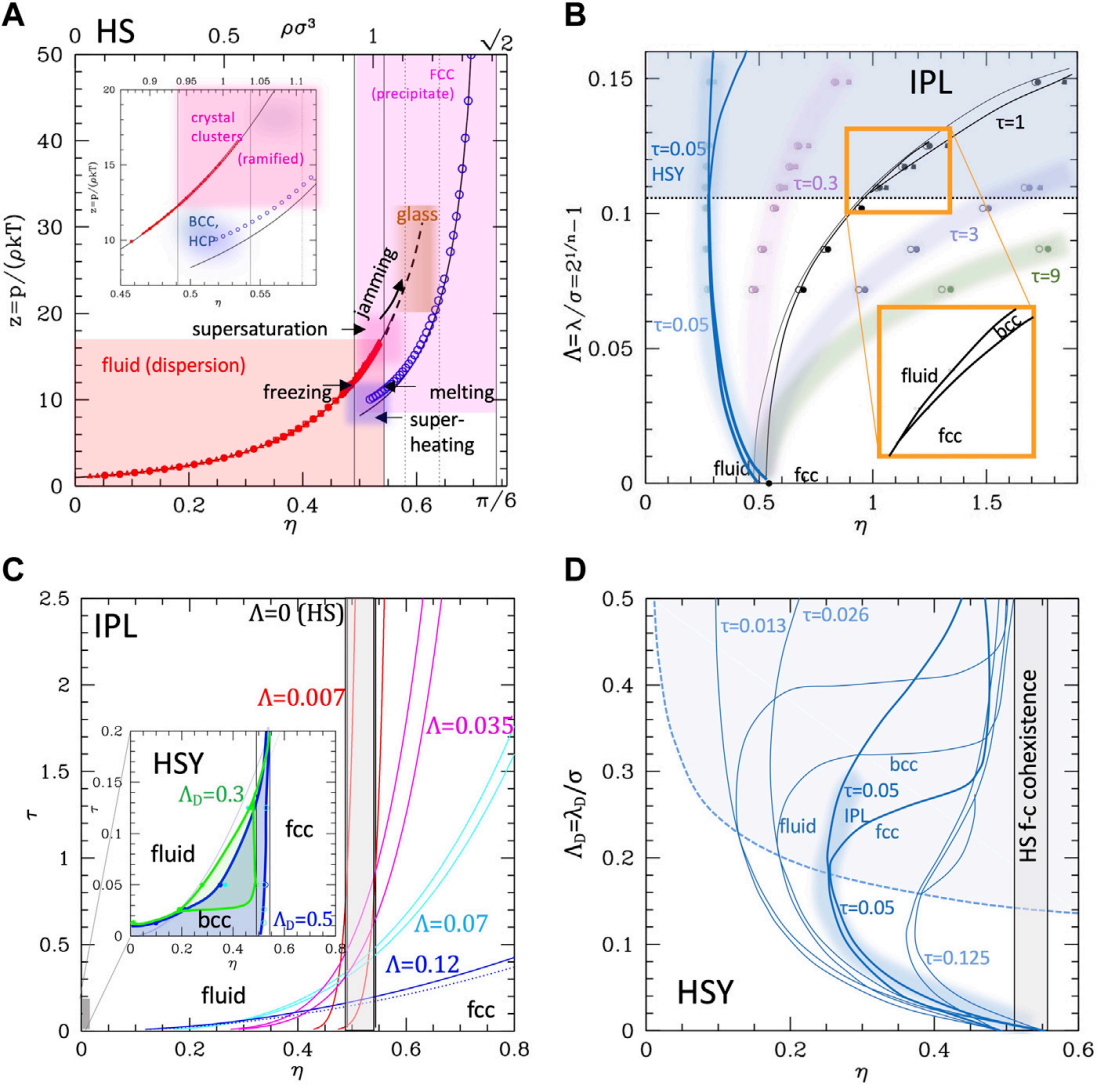
Phase diagrams of the repulsive-only systems. **(A)** Phase diagram and *z-η* EoS of the HS system, as a function of the packing fraction. Black lines: CS formula (Eq. 2) for the fluid branch, and WS formula (Eq. 3) for the FCC branch (Ustinov, 2017). The vertical solid lines are located at the melting and freezing packing fractions from refs (Erpenbeck and Wood, 1984; Hoover and Ree, 1968; Ustinov, 2017). The vertical dotted lines are the limits of the glass phase from refs (van Megen and Underwood, 1993; Rambaldi et al., 2006; Noya et al., 2008; Zykova-Timan et al., 2010; Pieprzyk et al., 2019; Luo and Janssen, 2020). The inset is a zoom into the coexistence region. The limits of the supersaturated and ramified cluster regions are taken from refs (Parisi and Zamponi, 2005; Anikeenko and Medvedev, 2007; Pusey et al., 2009; Sanz et al., 2011; Valeriani et al., 2012; Mulero and Tian, 2013; Wang et al., 2018), while the limits for the super-heated region and the approximate location of the metastable crystal phases BCC and HCP are taken from ref (Grimvall et al., 2012). **(B)** The phase diagram of the IPL system in the Λ-*η* plane (Λ= reduced range, see text), at different values of the reduced temperature. The black dots are taken from ref (Prestipino et al., 2005) for τ = 1 and connected with lines; dots and lines at different τ are calculated from the scaling law (Eq. 6). The triple point region is zoomed in. **(C)** The τ-*η* phase diagram of IPL at given values of Λ (values reported, colored with the same color of the corresponding curves, HS case is returned with Λ = 0). The melting and crystallization curves are reported as solid lines, enclosing the coexistence region; the dotted line visible only in the Λ = 0.12 case is the BCC–FCC transition line. The inset reports similar curves for the HSY case at given values of the Debye Length. **(D)** Λ_D_ *-*η phase diagram of the HSY system (Hynninen and Dijkstra, 2003) (reduced temperature reported). The same data are used to build the inset of panel c. The blue shaded area is the BCC phase existence region, while the shaded line is the IPL at the corresponding temperature τ = 0.05. (Data in numerical form are extracted from the reported refs and plotted.)

The phases of the HS system as a function of *ρ* (or *η*) have been widely investigated in the last century, both theoretically and by simulations, starting from the first study by van der Waals, based on the second virial coefficient. Since then, over hundred analytical expressions for the equation of state (EoS) were given, either based on approximate closure theories [reviewed in Mulero et al., 2001] or on the inclusion of the accurate values of the largest possible number of calculated virial coefficients (Clisby and McCoy, 2004; Bannerman et al., 2010). One of the most used analytical forms is the Carnahan Starling (CS) (Carnahan and Starling, 1969)

$$z\left(\eta \right)=\frac{1+\eta +\eta^{2}-\eta^{3}}{{\left(1-\eta \right)}^{3}}$$ (2)

(Figure 1A), which, though including only up to 4th virial coefficient, is quite simple, yet accurate at least in the region of stability of the fluid phase. At larger densities, in proximity of the freezing region, most of the simpler analytic expression bring large errors (e.g., Eq. 2 has an unphysical pole at *η* = 1). Formulas accurate up to the freezing or even in the coexistence region with the solid were obtained including higher virial coefficients at the expenses of the analytical simplicity (Mulero et al., 2001).

Because of contact-only nature of the interactions, the free energy per particle in HS is dominated by the entropy, depending on the accessible volume. This concept is exploited to evaluate the free energy (and *z*) for the crystalline solids: it is assumed that the accessible volume to a particle is basically the Wigner–Seize (WS) cell volume corrected by the volume occupied by the spheres, leading, for the FCC crystal, to the simple expression (Velasco et al., 1998)

$$z\left(\eta \right)=\frac{1}{\left(1-{\left(\frac{\eta }{\eta_{c}}\right)}^{\frac{1}{3}}\right)}$$ (3)

Using the CS and WS free energies, one gets 0.492 and 0.555 as the freezing and melting packing fractions, respectively (Wu and Prausnitz, 2002), clearly defining a first-order phase transition and a fluid-crystal phase coexistence region. This transition has been widely investigated by means of computer simulations for more than 60 years (Alder and Wainwright, 1960; Adams, 1974; Woodcock, 1976; Erpenbeck and Wood, 1984), with results nicely superimposing to the theoretical lines (Hoover and Ree, 1968), and bringing only small corrections to the limits of the coexistence region, whose values were recently established at *η*_*f*_ *∼* 0.491 (*ρσ* ^*3*^ = 0.938) and *η*_*m*_ *∼* 0.543 (*ρσ* ^*3*^ = 1.038), respectively (Ustinov, 2017) (Figure 1A).

It should be kept in mind that when HS represents colloids, the fluid phase corresponds to the disperse state, the solid one to a precipitate; the coexistence region is the most interesting, for the possibility of the formation of clusters. Simulations show that the fluid (dispersed) branch can exist at least up to the middle of the coexisting region (Zykova-Timan et al., 2010), and even beyond the melting density as a metastable “supersaturated” fluid (Pieprzyk et al., 2019), and vice versa, the FCC phase can extend below the melting (Noya et al., 2008) as a “superheated” crystal. Along the fluid branch, the mean free path continuously decreases (Rambaldi et al., 2006) and the dynamics slows down indicating a glass transition at η∼0.56–0.58 (van Megen and Underwood, 1993; Luo and Janssen, 2020). The maximum packing fraction for the amorphous solid was evaluated to be η∼0.64 (Richard et al., 1999; Parisi and Zamponi, 2005). The very nature of the glass transitions in the HS system is still under debate (Pusey et al., 2009), but the behavior of the system in the critical regions was explored by simulations. In spite of the absence of attractive interaction, in the super-saturated region, crystalline clusters form as nucleation centers, which can assume ramified or fractal structures for η> 0.54–0.56 (Valeriani et al., 2012), while in the glass-forming region, the formation of crystallites in the disordered phase appears kinetically hindered (Sanz et al., 2011; Anikeenko and Medvedev, 2007).

Conversely, descending to low η along the crystal line, the metastable crystal can exist (Mulero and Tian, 2013) down to a limit recently located at η∼0.494 (Wang et al., 2018). Around this value, the melting from FCC changes from a homogeneous nucleation to a “catastrophic” transition. Although the body-centered cubic (BCC) phase appears in the “superheated” conditions (Tejero and Cuesta, 1993), simulations and calculations agree that for the ideally HS, the fluid–solid transition occurs from the FCC without passing through a BCC intermediate, which turns out unstable under shear deformations (Grimvall et al., 2012). In fact, simulations started from the BCC phase for densities larger than the melting one exhibits the transition to FCC passing through metastable phases with HCP and FCC domains, while between melting and freezing densities, the transition occurs through the formation of BCC domains slowly relaxing to FCC–fluid coexistence. However, in order to observe these transitions, one must always start from a system in which the BCC crystal is stable, e.g. from soft spheres (see the next section).

### 2.2 Effect of the repulsive range: Soft spheres and screened electrostatics

The simplest possible perturbation to the HS system is the inclusion of a range in the repulsion. This needs at least one additional parameter to describe the range of the repulsion, λ (or *k* = 1/λ). One typical form used for the “soft sphere” model is the inverse power law (IPL) potential

$$u\left(r\right)=\varepsilon {\left(\frac{\sigma }{r}\right)}^{n}$$ (4)

This potential can describe weakly interacting soft colloids since the functional form also includes softness, together with the repulsive tail. Conventionally, the range is defined as the distance at which the potential is halved with respect to the value at the particle surface. Using this definition, for the IPL potential one gets ${\text{λ}}_{1/2}=\sigma \left(2^{1/n}-1\right)$ , if the range is measured from the surface (or $\text{λ’}={\text{λ}}_{1/2}+\sigma =\sigma 2^{1/n}$ if measured from the center). Conversely, for hydrophilic colloids, the potential must account for the Coulomb interaction and for the ionic screening. For polar ones, it is common to use the Yukawa potential with repulsive wall (HS Yukawa, HSY)

$$u\left(r\right)=\{\begin{array}{c} \varepsilon \frac{{\text{e}}^{-\kappa \left(r-\sigma \right)}}{r/\sigma }=k_{B}T\lambda_{B}Q^{2}\frac{{\text{e}}^{-\frac{r}{\lambda_{D}}}}{r}\;\;\;r>\sigma \\ \;\;\;\;\infty \;\;\;\;\;\;\;\;\;\;\;\;\;\;\;\;\;\;\;\;\;\;\;\;\;\;\;\;\;\;\;\;\;\;\;\;\;\;\;\;\;\;\;\;\;\;\;\;r<\sigma \end{array}\;$$ (5)

where *Q* is the effective charge of the particle, $\lambda_{B}=e^{2}/\left(4\pi k_{B}T\varepsilon_{r}\varepsilon_{0}\right)$ is the Bjerrum length, and the Debye screening length $\lambda_{D}=1/\kappa$ is naturally defined as the range, although at a distance $\lambda_{D}$ from the particle surface, the potential is decreased by a factor $\;\left(\lambda_{D}/\sigma \right)e$ *.* The HS limit is recovered when the range vanishes, i.e., n→∞ for the IPL potential (Eq. 4) and κ→∞ for HSY potential (Eq. 5); however, at variance with IPL, HSY does not include softness, preserving the hard-core repulsion. The IPL potential is often considered as a convenient regularized alternative to the HS (Grimvall et al., 2012), while the repulsive tail of HSY can be derived on physical bases within the linearized Poisson–Boltzmann (PB) approach (Denton, 2010a), returning explicit values for the parameters. In particular, the Debye length $\lambda_{D}=\varepsilon_{0}k_{B}T/\left(e\sqrt{8\pi \left(n_{+}+n_{-}\right)}\right)\;$ inversely depends on the ion concentration: the larger the ionic strength, the stronger the screening and the shorter the repulsive tail. The effective charge $Q=Ze^{\sigma k/2}/\left(1+k\sigma /2\right)\;$ is different from the intrinsic charge *Z* (Z in turn is the bare charge plus the charge due to possible counterion stably bound to the particle and which do not enter the screening density n±) of the colloids and accounts for the effective counterion-mediated colloid interactions, at least within the linear screening approximation and for low colloid densities. The inclusion of many body effects and counterion correlations at higher level is very complex, but can be achieved still using within a renormalization approach consistently re-scaling charges, ion densities, and colloid radii (Denton, 2010b). It is customary to assume a Derjaguin–Landau–Verwey–Overbeek (DLVO) potential and fit experimental data adjusting effective charge, which normally turns out considerably smaller than the surface charge determined from titrations. A simple method for calculating renormalized charges is still missing (Quesada-Pérez et al., 2002).

The range of the repulsive tail, often described by the dimensionless parameter λ/σ=1/κσ=Λ *,* produces several effects. The transitions are no more purely entropically driven; hence, the phase diagram will depend on the temperature *T*. Therefore, a more complex phase behavior is expected, with the possible appearance of new phases. Indeed, the BCC phase was experimentally observed in charged soft colloids with range comparable or exceeding their size (Kanai et al., 2015). For the IPL potential, there are additional symmetries: the thermodynamic behavior can be expressed as a function of a single dimensionless parameter (Verma and Ford, 2011) dependent on a combination of the reduced temperature τ=kT/ε and of η

$$\gamma =\frac{\rho \sigma^{3}}{\tau^{\frac{3}{n}}}=\frac{6}{\pi }\frac{\eta }{\tau^{\frac{3}{n}}}=\frac{6}{\pi }\eta {\left(\frac{\varepsilon }{k_{B}T}\right)}^{\frac{3}{n}}$$ (6)

that is a temperature-scaled density. HSY does not display the scaling properties, being a combination of two different functional forms; however, the related point-like Yukawa (PY) without repulsive wall does. In fact, for PY, the role of γ is taken by another dimensionless parameter

$$\text{Γ}=\frac{Q^{2}}{k_{B}T4\pi \epsilon_{0}a}=\frac{Q^{2}}{k_{B}T4\pi \epsilon_{0}}{\left[\frac{4\pi \rho }{3}\right]}^{\frac{1}{3}}=\frac{\eta^{\frac{1}{3}}}{\tau }{\text{e}}^{\frac{1}{{\text{Λ}}_{D}}}$$ (7)

*a* being the radius of the sphere occupied by a single particle. Indeed, for n = 1 in IPL and for ${\text{Λ}}_{D}=\infty$ in PY, one gets the equivalent relations for (6) and (7), i.e. $\gamma \propto \rho /T^{3}$ and $\text{Γ}\propto \rho^{1/3}/T$ , which is the well-known scaling law for the one component plasma (Martynova and Iosilevskiy, 2015), since both IPL and PY return the Coulomb potential in the long-range limit. HSY does not, preserving the hard-core repulsion. On the other hand, IPL and HSY return the HS limit for null range, while PY doesn’t. In general, missing a characteristic length describing the colloid size, PY is not a good representation of a potential for colloids and is considered here only as a support to interpret the phase diagrams of the others.

The phase diagrams of these systems are reported in Figure 1, panels b–d. The scaling law of IPL allows simulating the system at, e.g. τ=1  and then extending the results to other temperatures. Panel 2) reports the phase behavior in the plane $\eta -\Lambda_{1/2}\;\;$ (with $\Lambda_{1/2}=\lambda_{1/2}/\sigma =\left(2^{1/n}-1\right)$ ) for the IPL (data of ref (Prestipino et al., 2005) for τ = 1, black dots and lines). The abscissa axis ( $\Lambda_{1/2}=0$ ) corresponds to the HS system, with the fluid–FCC transition and coexistence region, as previously shown. As the range increases (i.e. moving vertically in the plane), however, the coexistence region becomes thinner and disappears for $\Lambda_{1/2}>$ 0.13. Additionally, when $\Lambda_{1/2}$ >0.1–0.12 (or n < 6–8) (Agrawal and Kofke, 1995), a region of stability of the BCC phase appears between the fluid and FCC phases, with a first-order transition between BCC and FCC (see the inset of Figure 1B). Using Eq. (6), one can obtain the phase diagram at different *τ*, shown with colored dots and shaded lines in Figure 1B (values of *τ* reported). The fluid–solid transition lines are not vertical: softness allows transition values of *η* to move to larger values due to penetrability of spheres, and the effect is larger as $\Lambda_{1/2}$ and/or τ increase. The effect of softening is increased at higher temperatures as the transition lines bend more, moving the melting–freezing and BCC–FCC transition to larger densities. Conversely, as τ decreases, the transition lines bend toward lower densities, decreasing the accessible range of packing fraction to the fluid and narrowing the coexistence region as $\Lambda_{1/2}$ increases.

The same information is reported in the τ*–η* (temperature–density) phase diagram in Figure 1, panel c (main plot), for different values of $\Lambda =\Lambda_{1/2}$ (reported). At vanishing values of the range, the HS vertical transition lines are returned (black), while as Λ increases, they lean to the right, extending the fluid region at high temperatures. Also relevant is the behavior at low temperatures: the region of stability of the fluid disappears in favor of the solid phases. This results in the region of stabilization of the BCC phase enlarging at low densities, especially for HSY (Rascón et al., 1997) (see the inset of panel c). The range-density phase diagram of HSY (Hynninen and Dijkstra, 2003) is reported in Figure 1D. Qualitatively, it is similar to IPL, in that the BCC phase appears for large values of ${\text{Λ}}_{D}=\lambda_{D}/\sigma$ as intermediate between the FCC and the fluid. Also, the bending of transition lines as a function of the temperature is similar, and the correspondence can be made quantitative once recognized that the halving range in IPL, *λ = λ*_*1/2*_ *,* and the Debye length *λ*_*D*_ are not exactly corresponding quantities, the latter being the distance at which the potential is decreased by a factor *e* (1+ *λ*_*D*_ */σ*). The general relationship between λ and λ_D_ is derived in the SI, Supplementary material S1, and is approximately *λ*∼ln (2)*λ*_*D*_ for short ranges. Once the quantitative relationship is used, the lines of phase separation for corresponding τ nicely superimpose (bold blue lines for HSY, shaded blue band for IPL, τ=0.05 ), at least at short average ranges.

However, at large ranges, the BCC phase appears wider in HSY, as anticipated. The specific shape of the phase diagram of this system can be better understood comparing with the point-like Yukawa (PY; see the SI, Supplementary Figure S2) displaying the typical “reentrant” form of the transition lines with a minimum value of *η* as the range is varied at fixed τ , and the typical enlarging of the BCC phase, the only one stable for the purely Coulomb solid. (The line of triple points between fluid, BCC, and FCC phase can be determined analytically in PY and works approximately also for HSY.) However, due to the presence of the impenetrable repulsive wall in HSY, at variance with PY, the BCC phase cannot exist up to infinite densities and must bend to adapt to the vertical transition to the FCC phase at large values of range, besides ending exactly to the *η*_*f*_ and *η*_*m*_ values as the range vanishes. This also implies that for intermediate and small-range values, the BCC phase shrinks disappearing to another critical point located at higher values of the range.

We can summarize as follows: 1) the softness produces the leaning of the fluid–FCC transition lines and coexistence region to higher densities as the temperature increases; 2) the repulsive range produces the stabilization and enlarging of the BCC phase at low temperatures, which basically substitutes the fluid phase at low densities. The two effects are present in the IPL potential, while only the second is present in HSY. It is important to remind that when ported to the colloid case, fluid means the completely dispersed phase. In addition, because the system is always kept at a constant volume (occupied by solvent), actually the crystal phases correspond to a complete separation and precipitation of the colloid at equilibrium. Clearly, the interesting conditions are those of coexistence or metastable transients, where (ordered) clusters and/or percolates can form.

### 2.3 Lennard-Jones and hard-core attractive Yukawa potentials

**FIGURE 2:**
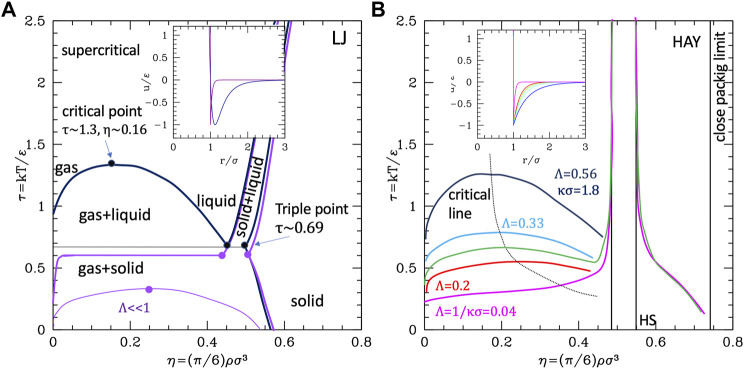
Phase diagrams of attractive potentials. **(A)** LJ phase diagram in the temperature–density plane (dark blue lines). The purple line represents the gas–liquid coexistence region for an LJ-like potential with shorter range (indicated). **(B)** Same for HAY at different values of the range (indicated). Data for the plots are extracted in numerical form from refs (Schultz and Kofke, 2018; Tuinier and Fleer, 2006).

Neutral colloids display an—usually weak—attractive well. In NPs, the degree of attraction can be modulated by the type of chemical functionalization. If the particle represents a globular protein, hydrophobicity is always present and variable. Therefore, an attractive tail must be added to the (soft) repulsive core. The most studied potential with these features is the Lennard-Jones (LJ), whose phase diagram is well known (Ge et al., 2003; Schultz and Kofke, 2018). In the original form (i.e. the 12–6 potential), it was used for the neutral and unpolar particles (such as noble gas atoms). At high temperatures (Figure 2A), LJ system displays the usual fluid-crystal first-order transition with transition lines leaning on the right due to the introduction of softness (by the -12 repulsion), while at a critical value of the temperature (τ_c_ = *kT*_*c*_ */ε* ∼ 1.3), a first-order transition between two disordered phases (namely gas and liquid) differing by the density appears, with a coexistence region that enlarges as the temperature decreases. Lowering further the temperature, one reaches the triple point (at τ ∼0.69), below which the liquid phase cannot exist and only a gas–solid broader coexistence region persists up to the crystallization density.

The stabilization of the condensed disordered phase is specifically due to the attractive well. In fact, the critical temperature depends on the well depth ε directly through the combination *kT*_*c*_ = τ_c_ */ε.* We observe that the LJ phase diagram can be regarded as the superposition of the “leaning” fluid–crystal transition region typical of the IPL potential (corresponding to the repulsive part of the potential) and a reversed parabola-like gas–liquid coexistence region due to the attractive part. Consequently, the coexistence region is expected to depend also on the range of attraction. LJ potential has a fixed range comparable with σ; therefore, in order to explore the dependence on range, the hard-core attractive Yukawa (HAY) (Valadez-Pérez et al., 2012; Tuinier and Fleer, 2006) potential was used

$$u\left(r\right)=\{\begin{array}{c} -\varepsilon \frac{{\text{e}}^{-\kappa \left(r-\sigma \right)}}{r/\sigma }\;\;\;\;\;\;\;\;\;\;\;\;\;\;\;\;\;\;\;\;\;\;\;\;\;\;\;\;\;\;\;\;\;r>\sigma \\ \;\;\;\;\infty \;\;\;\;\;\;\;\;\;\;\;\;\;\;\;\;\;\;\;\;\;\;\;\;\;\;\;\;\;\;\;\;\;\;\;\;\;\;\;\;\;\;\;\;\;\;\;\;r<\sigma \end{array}\;$$ (8)

(note that in this case the parameter kσ = 1/Λ is not related to a Debye length in this case but simply represents the range of the hydrophobic attraction). The possibility of varying—specifically reducing—the range makes HAY even more appropriate for colloids since due to their large size they have a generally relative range Λ shorter than LJ. An alternative to HAY is the generalized α-2α LJ whose range is, however, not straightforwardly related to the exponent α (see the Supporting Information, Supplementary Material S2), roughly ∼3/α for large α.

The temperature–density phase diagram for HAY is reported in Figure 2B. The reduction of the range is seen to have a similar effect to the reduction of the well depth: for small kσ—large Λ the system behaves similarly to standard LJ (except for the exactly vertical solid–liquid transition lines, due to hard core, in this case). For kσ∼1.8, the gas–liquid curve is quantitatively similar to LJ. However, as kσ increases (or the range decreases), the critical temperature lowers and the coexistence curve flattens, so to progressively reduce the region of stability of the liquid phase. This kind of behavior was observed to be independent on the specific kind of potential and is therefore similar in α-2α potentials: as Λ gets smaller than ∼0.15, the critical temperature falls below the triple point temperature (Lomakin et al., 1996). In these conditions, the gas–liquid coexistence region would lye entirely within the gas–solid coexistence region (see the purple line in panel a of Figure 2) and the liquid cannot exist as a thermodynamic stable state (Valadez-Pérez et al., 2012). When ported to the colloidal system, at high temperature, the system behaves qualitatively as the purely repulsive one, with a disperse-aggregate transition and a coexistence region described with the formation of ordered clusters and percolates. The “liquid gas coexistence” corresponds to the formation of disordered clusters, whose morphology is, interestingly, independent on the specific kind of potential (Soto-Bustamante et al., 2022). This phase is stabilized by the attractive part of the potential; therefore, for colloids with particularly weak/short-ranged attraction may not be present as thermodynamically stable phase. However, it is indeed reported in several works as a metastable condition, sometimes referred to as the “two phase region” (Liu and Xi, 2019), or even “liquid–liquid” coexistence (Wu and Prausnitz, 2002; Stradner and Schurtenberger, 2020a) below the critical temperature. If conversely the attractive part is stronger (as in type III, c colloids), the critical point rises and eventually the disordered clustered phase stabilizes (dark blue line in Figure 2, panel a).

**FIGURE 3:**
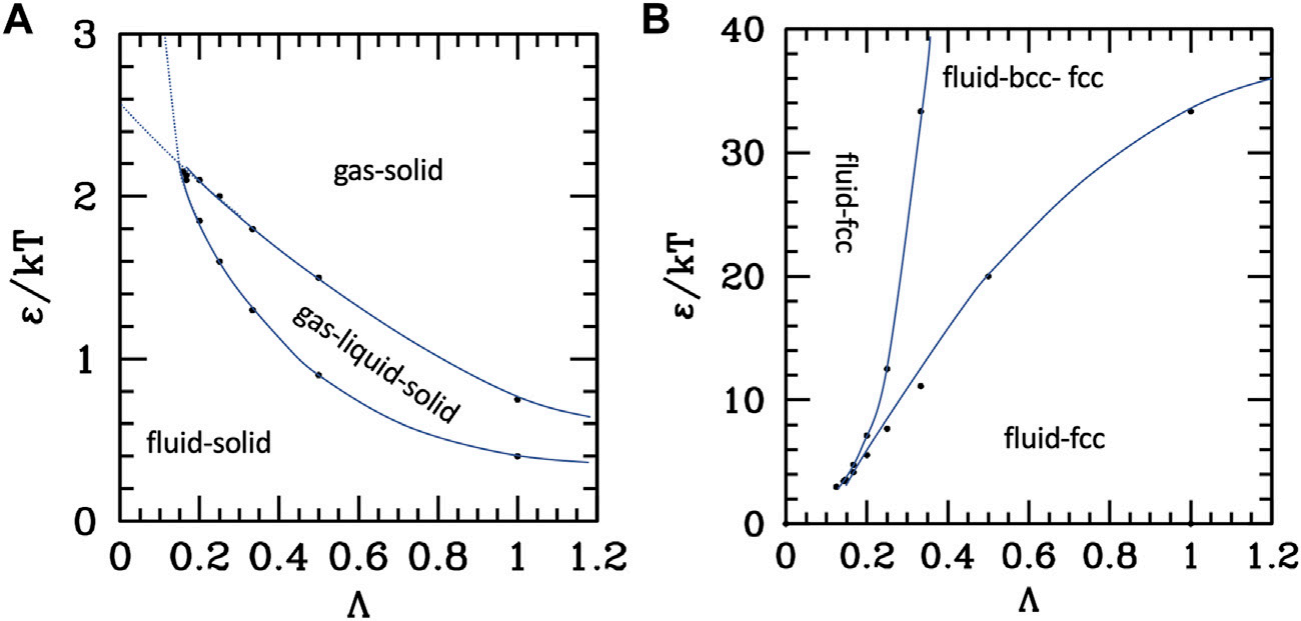
Phase diagrams of the attractive **(A)** and repulsive **(B)** potentials, as a function of the reduced range Λ = λ/σ and of the temperature renormalized strength ε/kT. Dots are numerical data extracted from (Tuinier and Fleer, 2006; Makuch et al., 2015), Blue lines are guide for the eye.

To summarize these results, we report in Figure 3 the parametric phase diagrams, using as parameters the inverse of the strength renormalized temperature 1/τ = ε/kT and the diameter renormalized range Λ. An expanded version of the parametric phase diagram as a function of the reduced temperature and inverse ranges is reported in the SI, Supplementary Material S3.

The data to build the curves are taken from works using the Yukawa (attractive (Tuinier and Fleer, 2006) or repulsive (Makuch et al., 2015)) plus a hard-core repulsion at *σ*; however, they are extensible to other attractive or repulsive potentials with similar characteristics (i.e., a repulsive core, a repulsion range, or an attractive tail with variable range). The attractive potential diagram in Figure 3A shows the fluid–solid (FCC) transition at high temperature (or small attraction) and small-intermediate range, and the appearance of the liquid phase in the long-range-intermediate temperature region. At very low temperature or intermediate temperature and long ranges, the liquid phase is destabilized in favor of the ordered phase, and the same happens at any temperature for very short ranges because the critical line (upper in the plots τ as the y axis) crosses and goes below the triple point line. However, the plots report the extension of those lines beyond the crossing (dotted), which identify the metastable phase coexistence region.

Conversely, without attraction (Figure 3B), the aggregated disordered phase does not exist; however, the BCC crystal can be stabilized at a large range and low temperature. Interestingly enough, for short ranges, attractive and repulsive systems behave similarly, i.e., with the fluid–crystal transition only, with fluid stability decreases as the range increases, therefore extending the coexistence region; in the case of repulsive potential, a part of this coexistence region is additionally occupied by the FCC phase.

## 3 Potentials with competing interactions

As far as other interesting systems, functionalized metal nanoparticles may display short-range attraction (due, e.g., to hydrophobic functionalization) and long-range repulsion (due to possible net charge). Different potentials (collectively named SALR, Short range Attraction, Long range Repulsion) were used to represent these conditions, some of them reported in Table 2. Generally, the potential is expressed as a simple additive form of a shorter-range attractive part using one of the previously described, plus a longer-ranged repulsive part generally described by a Yukawa form because the repulsive wall at σ is brought by the attractive component pure Yukawa can be used for repulsion.

**TABLE 2:** A summary of the properties of the different types of repulsive and attractive potentials.

Potential		Parameters and relationships	Refs
$u\left(r\right)=\varepsilon \left[4\left({\left(\frac{\sigma }{r}\right)}^{2\alpha }-{\left(\frac{\sigma }{r}\right)}^{\alpha }\right)+\;a\left(\frac{\sigma \xi }{r}\right)\;e^{-\frac{r}{\sigma \xi }}\right]$	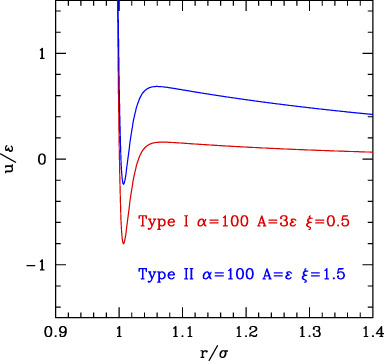	Diameter σ	Mossa et al. (2004)
		*α* = 100 → Λ_0_∼ 0.025	
		Attractive strength *ε*	
		Repulsive strength a *ε*	
		Repulsive range *σξ*	
		Type I or II	
$u\left(r\right)=4\varepsilon \left({\left(\frac{\sigma }{r}\right)}^{2\alpha }-{\left(\frac{\sigma }{r}\right)}^{\alpha }\right)+\;A\left(\frac{\sigma \xi }{r}\right)\;e^{-\frac{r}{\sigma \xi }}$	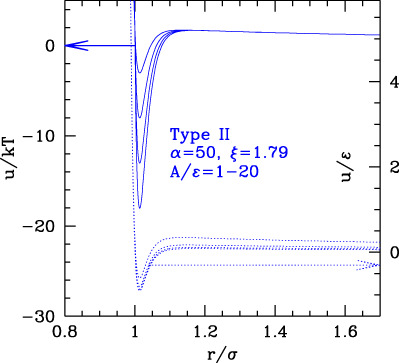	*α* = 50 → Λ_0_∼0.06	Mani et al. (2014)
		ξ=1.79	
		A=2kT	
		*ε*=1-20 kT	
		Type II	
		*α* = 12 → Λ_0_∼ 0.4	
$u\left(r\right)=\varepsilon \left[4\left({\left(\frac{\sigma }{r}\right)}^{2\alpha }-{\left(\frac{\sigma }{r}\right)}^{\alpha }\right)+\;a\left(\frac{\sigma \xi }{r}\right)\;e^{-\frac{r}{\sigma \xi }}\right]$	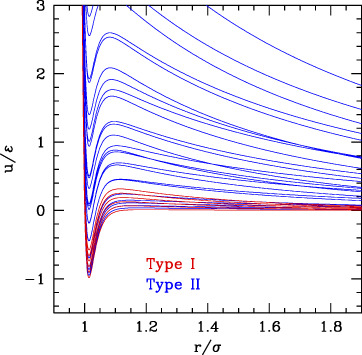	ξ = 50 → Λ_0_∼0.06	Bollinger and Truskett, (2016)
		ξ=0.7-4	
		A ∼[1-3]kT	
		[varied through the effective charge (Z)]	
		*ε*=1-6 kT	
$u\left(r\right)=A_{1}\left({\left(\frac{\sigma }{r}\right)}^{2\alpha }-{\left(\frac{\sigma }{r}\right)}^{\alpha }\right)+\;A_{2}\left(\frac{\sigma \xi }{r}\right)\;e^{-\frac{r}{\sigma \xi }}$	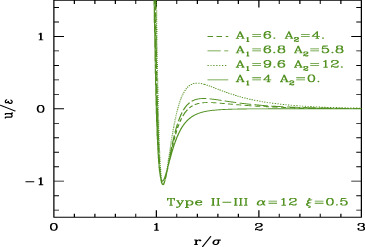	Attractive strength *A*_1_	Ioannidou et al. (2016)
		Repulsive strength A_2_	
		*α* = 50 → Λ_0_∼0.06	
$\frac{u}{kT}=\{\begin{array}{c} \frac{\sigma \;(-e^{-z1\left(\frac{r}{\sigma }-1\right)}\;+\;\lambda e^{-z2\left(\frac{r}{\sigma }-1\right)})}{\tau \left(1-\lambda \right)r}\;\;\;\;\;\;\;r>\sigma \\ \infty \;\;\;\;\;\;\;\;\;\;\;\;\;\;\;\;\;\;\;\;\;\;\;\;\;\;\;\;\;\;\;\;\;\;\;\;\;\;\;\;\;\;\;\;\;\;\;\;\;\;\;\;\;\;\;\;\;\;\;r<\sigma \end{array}$	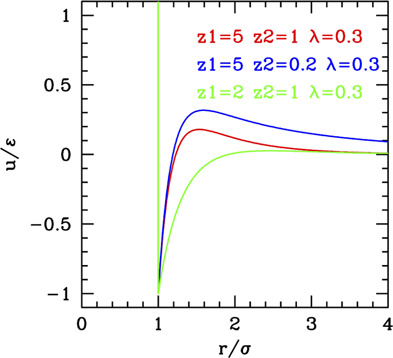	Reduced temperature τ	Godfrin et al, (2014)
		Inverse attraction range z1 = 1/Λ_0_	
		Inverse repulsion range z2 = 1/Λ_1_	
		Ratio between the strength of	
		repulsion and the attraction *λ*	

There were several attempts of classifications depending on the relative attractive ${\text{Λ}}_{0}$ and repulsive ${\text{Λ}}_{1}$ ranges, which are, however, not fully consistent. In ref (Liu and Xi, 2019), three classes are identified, namely, I, with attractive and repulsive short ranges ( ${\text{Λ}}_{0}$ <0.2, ${\text{Λ}}_{1}$ <1), II, with short attractive and large repulsive range ( ${\text{Λ}}_{0}$ <0.2, ${\text{Λ}}_{1}$ >1), and III, with long-range attraction and average range repulsion ( $0.2<{\text{Λ}}_{0}<1$ , ${\text{Λ}}_{1}∼1-2$ ). In Zhang and Liu (2014), however, while the III is still the class where the attraction is dominant, the role of I and II seems interchanged, I being the class with large repulsive range and II the one with short repulsive range. In other works, the phase diagram is studied keeping fixed the ranges and increasing the attraction–repulsion barrier (Valadez-Pérez et al., 2021), which, in fact, is also expected to influence the phase behavior. We observe that, especially in the simple additive form, the barrier of the resulting potential has a height that increases both with the intensity (A) and the range ( ${\text{Λ}}_{1})$ of the repulsive part, while it is always roughly located just after the attractive well, i.e., at a distance from the repulsive core, which is one or twice the attractive range, meaning that the effective “beginning” of repulsive tail is at $1+{\text{cΛ}}_{0}$ with c∼1–2. (An alternative form of the potential involving a switch function in place of a simple sum would give a better control of the barrier heigh and location, but it is barely used, because less manageable. Some comments on this are reported in the SI, Supplementary Table S2.)

In order to prevent ambiguity, here we adopt the following novel classification, in classes with increasing global weight of the attraction with respect to repulsion, either due to range or strength:

• Class a: short range/weak attraction and long range/strong repulsion (class II of (Liu and Xi, 2019 or I in Zhang and Liu, 2014).
• Class b: attraction and repulsion of comparable range/strength (class I in (Liu and Xi, 2019) or II in Zhang and Liu (2014)).
• Class c: larger range/strong attraction, average range repulsion (class III).

**FIGURE 4:**
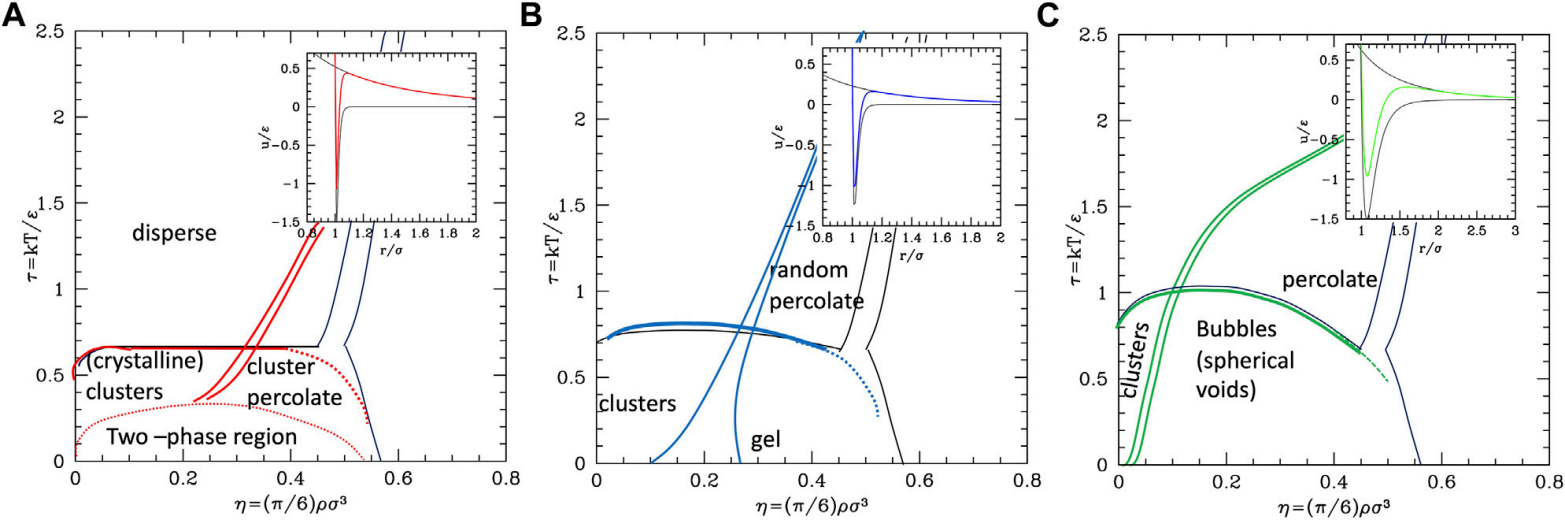
SALR ordered by increasing the relative weight of attractive to repulsive parts.

The three classes are depicted in Figure 4.

The phase behavior of SALR was studied in the last decades by different authors with different potential combinations. The most widely used to describe the short-range attraction is the 2α–α potential with α ∼ 50–100 (Bollinger and Truskett, 2016; Mani et al. 2014) for class a, 15–18 (Mossa et al., 2004) for class b, and 12 (Ioannidou et al. (2016)) for class c, combined with repulsive Yukawa (see Table 2). The parametric phase diagram using α = 100 in the plane $({\text{Λ}}_{1}$ , A/ε) (i.e., range and relative strength of the repulsion) was explored in Mossa et al. (2004) and compared with the attractive only-case with α = 6–18. At small values of ${\text{Λ}}_{1}$ and A/ε, they observe the formation of small spherical clusters resembling homologous of the droplets forming in the gas–liquid phase coexistence region. Conversely, for very large values of ${\text{Λ}}_{1}$ and A/ε, the completely dispersed phase tends to grow at the expenses of the liquid one, which can be understood considering the behavior of purely repulsive potentials. Interestingly, there is an intermediate region of parameters where the clustered phase displays linear or lamellar structures with a local crystalline order, which tend to grow and extend over the whole volume. Similar results were obtained in Mani et al. (2014), where the phase diagram of the SALR (α = 50, ${\text{Λ}}_{1}$ = 1.79 and A = 2 *kT*) was extensively explored by means of MD simulations at RT, varying the strength of attractive well in a wide range (Table 2, second row) and the density. The authors observe the usual disperse-FCC transition at low values of ε as η increases. As the attractive well increases, the colloidal cluster phase is observed, consisting of compact crystalline clusters, whose size increases with the colloid density, to eventually form a percolate extending over the whole volume (gel). Considering that both A and ε are expressed in units of *kT*, the ε–η phase diagram can be regarded as a τ–η phase diagram (with inverted vertical axis) with the repulsive part decreasing as τ increases. Therefore, the clustered phase can be interpreted as the homologous of the wide coexistence region observed in short-range attractive potential (Figure 2 panel a), within which the long-range repulsion drives a further transition between small clusters and percolated phase, also reported in earlier works (Verduin and Dhont (1995)). The phase diagram of the same potential was more recently explored varying both the attractive and repulsive relative strengths and ranges to extend further the exploration of SALR types a and b by means of simulations combined with liquid theories (Bollinger and Truskett, 2016). In this work, the clustered phase is identified by the presence of an intermediate-range order pre-peak in the structure factor and observed in a region extending toward large values of ${\text{Λ}}_{1}$ and of ε/kT. Later on, even the wider and stronger attractive range region was explored by simulations at a fixed well depth and variable barrier height (Ioannidou et al., 2016), so to explore the clustering-percolation transition for also type-c. Substantially similar results were obtained exploring the strong attraction region with a different potentials combining Yukawa repulsive with Yukawa attractive (Godfrin et al., 2014) (Table 2) by means of Monte Carlo Simulations and quantitatively identifying the phases by means of the size distribution of clusters. As in the previous case, the authors identify the dispersed fluid phase at high T and low density, the random percolated at high temperature and high density, the clustered fluid at low temperature and low density, and the clustered percolated at low temperature and high density.

The conclusion stemming from these works is that clusters with self-limiting size are stabilized in rather spherical and low symmetry forms by the competition between the attractive well and repulsive tail. As the density increases, the clusters tend to connect between each other forming a percolate, which can eventually occupy the whole volume as the density further increases. Accordingly, theoretical studies based on mean field theories showed the emergence of regions of excess density of variable size associable to clusters and identified the conditions, fulfilled by the SALR potentials, for micro-segregation (Ciach et al., 2013). A microscopic explanation of these disordered phases and methods for their possible identification has been discussed in a recent review (Ruiz-Franco et al., 2021). The “percolation transition” is observed in basically all types (a,b,c), but it can have a different character. Considering that it is due to the presence of the long-range repulsion, it may be regarded as the homologous of the fluid–solid transition line in the purely repulsive potentials, but shifted to about σ/(σ+2λ_0_) on the left, due to the fact that the long-range repulsion barrier is located roughly at (σ+2λ_0_); in addition, this line will be more leaning on the right due to the larger range of repulsion with respect to the hard-core one. This line crosses the gas–liquid (or dispersed-aggregate) coexistence region due to attraction, and therefore four main phases are identified in all cases: totally disperse and percolate above the critical temperature, clustered and percolated clusters below the critical temperature.

As the relative weight of repulsion to attraction changes, several variants of this scheme can appear. For temperatures lower than the barrier between attraction and repulsion, if the range of attraction decreases, the percolation line moves toward the right, to finally merge to the fluid–solid transition line (moving from c to a in Figure 4) turning in purely repulsive diagram. Before this, however, an additional effect can be observed: the very small attractive range can give rise to the metastable liquid–liquid coexistence region due to the falling of the critical temperature below the triple point line (panel a of Figure 4). Additionally, if the barrier becomes very high ( $∼20{\text{k}}_{B}T$ ) and attractive strength is also kept high ( $∼40{\text{k}}_{B}T$ ) while decreasing the range of attraction, metastable branched clusters can with a thin backbone and specific aggregate types such as Bernal spirals can form (Haddadi et al., 2021a).

Conversely, if the range or strength of repulsion decreases (or relative range/strength of attraction increases), the percolation line moves toward the left, so to finally disappear leaving the system to behave as purely attractive. In type c where the repulsion is still present though weak or short-ranged, besides the usual disperse and percolate phase, the presence of two periodically modulated phases (spherical clusters at low density and spherical voids at higher density) was identified below the binodal line (Archer et al., 2007) (Figure 4C) or rather below the λ line identifying the uniform-modulated fluid transition (Archer et al., 2008). In all intermediate cases (a and b), percolation line can cross the critical binodal line in different locations, generally at density smaller than the critical one. For this reason, in the studies on SALR, only a portion at small densities of the whole phase diagram is explored. The considered values of attractive range are generally not so small to make the two-phase region clearly visible as in Figure 4,a. However, an intermediate phase between clusters and percolate below the binodal line is often identified (Valadez-Pérez et al., 2021) (Figure 4B), which could also be related to the appearance of additional order due to the large range of repulsion, homologous to the appearance of the BCC phase in purely repulsive potentials. At high temperatures, the system is dominated by the repulsive core at σ , and the percolation line bends to the right (Valadez-Pérez et al., 2013), with more or less accentuated curvature depending on the strength of repulsion. Finally, the effects of the introduction of a second attractive well, for distances larger than the repulsive range, have been studied in Perdomo-Pérez et al. (2022). They found that this causes the binodal line to shift to higher temperatures, as in highly attractive systems. Interestingly, the increase of the second attractive well affects some physical properties in a way opposite to the increase of the repulsive strength, e.g., clusters tend to be more compact, while highly repulsive systems favor more elongated structures.

The described behavior is analogous to that observed for porous systems (Lindquist et al., 2017) where a different point of view is taken, with voids and filled spaces inverted. Even in this case, the formation of porous phases is found to be associated with SALR pair potentials. The repulsive strength and the attractive range are related to the pore diameter and the attractive strength to the packing fraction. Interestingly enough, the phase diagram obtained is qualitatively similar to that described above: for high attraction values, by decreasing the packing fraction, the pores merge forming ordinate spaces isolating clusters of particles with a preferred size, similar to those identified under the binodal line, while, for lower attraction strength [or by increasing the temperature (Lindquist et al., 2016)] spaces tend to be more unstructured until the void spaces form and percolate and isolated particles coexist with disordered clusters of different sizes.

We remark that, as for the interpretation of the Lennard-Jones phase diagram on the basis of the repulsive core + attractive tail, also in this case, the features of the SALR can be interpreted on the basis of the combination of those stemming from an attractive part and a repulsive one, both with variable range. A more detailed analysis of the possible combination of attractive and repulsive components including more cases than the SALR types I–III usually examined in the literature is outlined in the SI, Supplementary Figure S4.

## 4 Experimental realization of potentials with competing interactions

While purely repulsive potentials and weakly attractive soft-core potentials of Sections 2.2, 2.3 are commonly realized with charged and neutral nanoparticles, respectively, the SALR are often obtained in the case of charged core nanoparticles functionalized with hydrophobic groups. However, fine tuning the repulsive to attractive part is not straightforward experimentally. Recently, De Vivo and co-workers (Franco-Ulloa et al., 2020) made a step forward in this direction showing how the interaction between the metallic core NPs can be tuned to reach the desired pair potential. They estimated zeta potential (i.e., the potential at the interface between the mobile ions and dispersant) of citrate-covered gold NPs using coarse-grained-MD simulations as a function of two parameters: the surface charge density (σ) of the NPs and the ionic strength of the medium (I). By mapping the zeta potential of all systems into a bidimensional plot with contour delimiting values of the surface charge σ and salt concentration I, it was possible to separate colloidal stability vs. instability, comparing the theoretical data with aggregation in experiments. Also, the well depth of the van der Waals interaction can be modified by calculating the free energy of dimerization of the model NPs and this can be compared with experiments, e.g. the computed dispersion state phase diagram of citrate-coated metallic nanoparticles in saline solutions can be compared with ultraviolet–visible spectroscopy experiments to validate the theoretical predictions.

NPs interacting with SALR potentials can also be realized by grafting hydrophobic surface layers onto charge-stabilized particles, e.g. polyethylene glycol-grafted polystyrene particles (Haddadi et al., 2021b). The relevant parameters of this potential can be experimentally tuned. The repulsive strength (A) is related to the charge of the particles, which can be due to the surface functionalization or to the ionic specific absorption, while the repulsive range $\left({\text{Λ}}_{1}\right)\;$ can be lowered, passing from class a to class b systems by adding salt to the solution. The effective charge of the particle and the effective range of the repulsion are tunable through the measured Zeta potential (Van Gruijthuijsen et al., 2013). The repulsive barrier height and location can also be tuned by modifying the density and the length of the polymer chains (Guo et al., 2020), measurable with small-angle neutral scattering. Finally, attraction can be produced by a hydrophobic core (e.g., PS core) or by the addition of nonadsorbing polymer whose dimension and concentration control the attractive strength and range (Campbell et al., 2005; Klix et al., 2010). However, there are some limitations in experiments. It is difficult to keep the ionic strength very low to obtain a range of repulsion of the order of particle size (as in SALR a and c potentials). One possibility is to decrease the particle size, though still remaining in the colloid range. Additionally, the range of attraction depends in a non-trivial way on the temperature of the system due to possible structural transitions of the polymers. Computer simulations are a valuable tool to account for all of these effects, which can affect the aggregation phase diagram.

## 5 Summary, conclusions, and perspectives

The phase diagrams of the colloids are often explored with theoretical approaches using extremely simplified potentials, isotropic in first approximation and with implicit solvents. In these conditions, they are homologous of single component fluids; however, where the gaseous phase corresponds to the fully dispersed one, the solid to precipitate and the liquid and possible coexistence phase can be put in correspondence with the variety of colloidal phases. With respect to simple fluids, however, the interaction potential of colloids includes some specificities, such as the large size (and consequently the relative short order of interaction) and, especially, double feature of short/average range attraction and longer-range repulsion (in the charged case), which enrich the phase diagram.

In this work, we have perspectively revisited the phase behavior of isotropic potentials with increasing complexity, illustrating how the additional phases appear as specific features are added to the potential. Starting from the HS potential describing with poor realism weakly interacting colloids, with only a disperse-precipitate transition with a coexistence region, we have shown that the introduction of softness includes the dependence on the temperature of the transition, while the introduction of the repulsive range stabilizes the BCC form of aggregates besides the FCC one in the coexistence. Adding an attractive well to emulate the hydrophobicity stabilizes the clustered phase, which is metastable; however, if the range of attraction is very short with respect to the size of the colloidal particle. Adding the repulsive tail self-limits the size of the clusters, additionally introducing a further percolation transition line whose location and temperature dependence are strongly modified by the relative weight of the repulsion with respect to attraction. This analysis allows us to clarify the different behavior of colloids belonging to the different classes defined in the literature and possibly to further explore cases not previously considered.

The competitive potentials can be used to treat the already mentioned metal-functionalized nanoparticles, where the short-range attraction is due to the possible hydrophobic functionalization and the long-range repulsion to the possible presence of a net charge. However, soft, weakly attractive and SALR potentials were also considered to represent aggregation behavior in proteins, especially the globular ones, already for several decades (Noro and Frenkel, 2000), and shown to catch the fundamental features of aggregation transitions even in those very complex systems. It is beyond the scope of this work to analyze in detail these aspects, but we remark that proteins have generally not isotropic interactions due to irregular form and charge distribution. Therefore, the use of isotropic potentials brings some limitations. As a consequence, proteins tend to display a considerably richer diversity of phases and self-organization behavior, typical of elliptical, patching colloids, appearing in different ranges of temperature and concentration and especially appearing when the diffusive or collective dynamical behavior is analyzed. During the last two decades, there has been a rapidly increasing theoretical and simulation effort to report on the study of anisotropic, patchy, and/or responsive colloids in which the particles can interact via directional and specific interactions, thus starting to resemble their complex biological counterparts. There is obviously an enormous potential for the application of these new concepts to protein solutions, and these aspects were recently reviewed in Stradner and Schurtenberger, (2020b).

In this work, we tried to focus on the global phase behavior as a function of physical parameter such as relative ranges/strength of attraction/repulsion, rather than on the parameters of the specific potentials, so to be able to identify the main physical determinants of aggregation, and to expand the possibility of using these potentials as a complementary tool to augment experimental studies that aim to design protein–nanoparticle interactions. In fact, in these simplified forms, the colloidal potentials can be easily used in simulations to represent the behavior of a mixture of the two systems, with a potential in many areas as pharmaceutical formulation (therapeutic effect of NPs as anti-aggregants) and materials sciences. An essential point in the application of concepts from colloid physics to protein–nanoparticle systems, however, is that it requires a case-by-case analysis on the level of coarse graining needed for a given problem and a critical choice of the experimental techniques and data chosen for a meaningful test of model predictions. Clearly, in this respect, a large number of actions can be pursued to improve the models in the sense of realism. One possibility is the inclusion of the anisotropic and patchy nature of colloids to describe protein interactions and/or to account of uneven distribution of the functionalization of NPs. Alternatively, a possibility is to add secondary smaller spheres on the surface of the primary sphere to account for the roughness of the NP surface, and the specificity of the chemical functionalization, which plays crucial roles in the interaction with proteins. This approach has been proposed recently by our group since it allows us to introduce a double scale representation of the system, (Brancolini and Tozzini, 2019a; Brancolini and Tozzini, 2019b) with the advantage of preserving the isotropy of each interacting unit, thus leading to a simpler implementability of the model into simulation codes.

The present review article has attempted to critically discuss the exploitation of colloid science concepts to better understand and predict the phase behavior of functionalized nanoparticles and/or protein–nanoparticle mixtures. We believe that with the colloid approach, we can drive forward the field with concepts that are underpinned by the molecular scale insight derived from models and that can be further tested and refined by confrontation with experimental reality to generate technologies with enormous societal impact.
