# Supplementary material for: Aggregation behavior of nanoparticles: Revisiting the phase diagram of colloids
Source: Front Mol Biosci. 2022 Sep 19;9:986223. doi: 10.3389/fmolb.2022.986223 (PMC9527328; doi:10.3389/fmolb.2022.986223)
Supplement: Supplementary file 1 [file DataSheet1.PDF]

Supplementary Material to

Aggregation behavior of bio-nanoparticles: revisiting the phase diagram of colloids with competing interactions

Margherita Bini[1], Giorgia Brancolini[2*], Valentina Tozzini[1]

[1] Istituto Nanoscienze – Cnr, Lab NEST SNS, Piazza San Silvestro 12, 56127 Pisa Italy

[2] Istituto Nanoscienze – CNR, Center S3, via G. Campi 213/A, 41125, Modena, Italy

*** Correspondence:** giorgia.brancolini@nano.cnr.it

S.1 Comparison of ranges of IPL and HSY

A comparative table of the repulsive only potentials cited in the main text is reported in Table S.1. Useful relations and plots are also given. Some of these need a further explanation. In the main text we defined the halving range of the interaction as the distance from the sphere surface where the potential is halved with respect to the one at the surface. For the IPL potential this leads to

$$\frac{\lambda_{1/2}}{\sigma} = \frac{1}{k\sigma} = \Lambda_{1/2} = 2^{1/n} - 1 \tag{S.1}$$

For the HS-Yukawa potential the range is usually identified with the Debye length λ_D. However, at distance λ_D from the surface, the HSY potential is $1/(e(1+\lambda/\sigma))$ of the value at the surface. In general the relationship between λ_D and $\lambda_{1/2}$ in HSY is

$$\frac{\lambda_D}{\sigma} = \Lambda_D = \frac{\Lambda_{1/2}}{\ln(2) - \ln(1 + \Lambda_{1/2})} = \frac{\lambda_{1/2}/\sigma}{\ln(2) - \ln(1 + \lambda_{1/2}/\sigma)} \tag{S.2}$$

(S.2) is a function $\Lambda = f(\Lambda_D)$ (or $\lambda = f(\lambda_D)$) reported in Figure A.1. At small values of λ_D the relationship is linear $\lambda_{1/2} \sim \lambda_D \ln(2)$ (dotted line), while for large values of λ_D the halving range saturates at $\lambda \sim \sigma$.

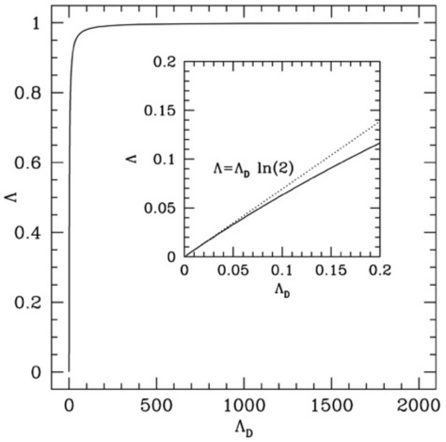

Supplementary Figure S.1 The relationship between the Debye length and the halving range of the interaction in HSY potential.

S.2 Comparison of parameters of repulsive potentials

In table S.1 the plots of HSY are reported at the values of the reduced Debye length given in the legend. For IPL, plots are reported for non integer values of n (as in the legend), corresponding to the reported halving length, which in turn corresponds to the same color Debye lengths of HSY. The two sets of potentials looks in fact quite similar, except for the fact that HSY is hard repulsive wall at r=σ while IPL display softness. In the case of PY potential, σ is no more a parameter of the potential, therefore the distances are referred to the Bjerrum length $e^2/4\pi\epsilon_0 k_B T$. If the curves are further renormalized with a the factor e^{1/Λ_D} they align to HSY for large r, while display soft behavior for large values of the range as IPL. However, as for null value of the range (i.e. $\Lambda_D = 0$ and n=∞ $\Lambda_{1/2} = 0$) PY has a different behavior from IPL and HSY. In fact, these return exactly the HS system, while PY vanishes; correspondingly the dimensionless parameter γ in IPL is well behaved, becoming a simple function of η only, while the corresponding parameter in PY, Γ, diverges. This is an effect that PY describes point-like particles, with no size, therefore if also the range vanishes, the interaction is ill defined.

	Potential	Formula	Parameters	Plots
**IPL**	Inverse Law Power	$u(r) = \varepsilon \left(\frac{\sigma}{r}\right)^n$	$\eta = \frac{\pi}{6}\rho\sigma^3 \quad \tau = \frac{k_B T}{\varepsilon}$ $\Lambda_{1/2} = \frac{\lambda_{1/2}}{\sigma} = 2^{1/n} - 1$ $\gamma = \frac{\rho\sigma^3}{\tau^{\frac{3}{n}}} = \frac{6}{\pi}\frac{\eta}{\tau^{\frac{3}{n}}} = \frac{6}{\pi}\eta\left(\frac{\varepsilon}{k_B T}\right)^{3/n}$	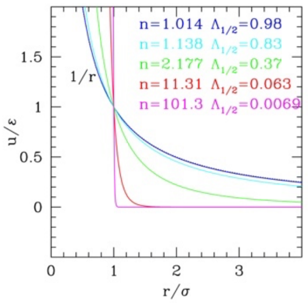
**HSY**	Hard Sphere Yukawa	$u(r) = \begin{cases} \varepsilon \frac{e^{-\kappa(r-\sigma)}}{r/\sigma} & r > \sigma \\ \infty & r < \sigma \end{cases}$	$\eta = \frac{\pi}{6}\rho\sigma^3 \quad \tau = \frac{k_B T}{\varepsilon}$ $\Lambda_D = \frac{\lambda_D}{\sigma} = \frac{1}{\kappa\sigma}$ $\Lambda_D = \frac{\Lambda_{1/2}}{\ln(2) - \ln(1 + \Lambda_{1/2})}$	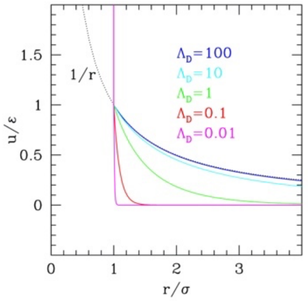
**PY**	Pointlike Yukawa	$u(r) = k_B T Q^2 \frac{e^{-(\frac{r}{\lambda_B})/(\frac{\lambda_D}{\lambda_B})}}{r/\lambda_B}$ $= \frac{Q^2}{4\pi\epsilon_0}\frac{e^{-r/\lambda_D}}{r}$	$\frac{Q^2}{4\pi\epsilon_0} \xrightarrow{\text{PY→HSY}} \varepsilon\sigma e^{\sigma/\lambda_D}$ $a = \left[\frac{3}{4\pi\rho}\right]^{1/3} \xrightarrow{\text{PY→HSY}} a = \frac{\sigma}{2\eta^{1/3}}$ $\Gamma = \frac{Q^2}{k_B T 4\pi\epsilon_0 a} = \frac{Q^2}{k_B T 4\pi\epsilon_0}\left[\frac{4\pi\rho}{3}\right]^{\frac{1}{3}}$ $\Gamma \xrightarrow{\text{PY→IPL/HSY}} \frac{\eta^{\frac{1}{3}}}{\tau} e^{\sigma/\lambda_D}$	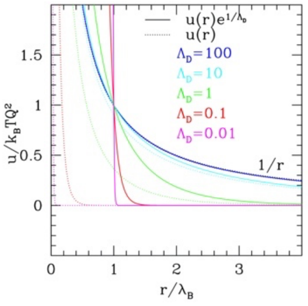

Supplementary Table S.1. Comparative table of repulsive only potentials considered in this work. Formulas, relevant parameters defining density, range and dimensionless scaling laws are reported. Representative plots are reported. For PY the correspondence with HSY and IPL parameters are reported, although some of them are ill defined for PY.

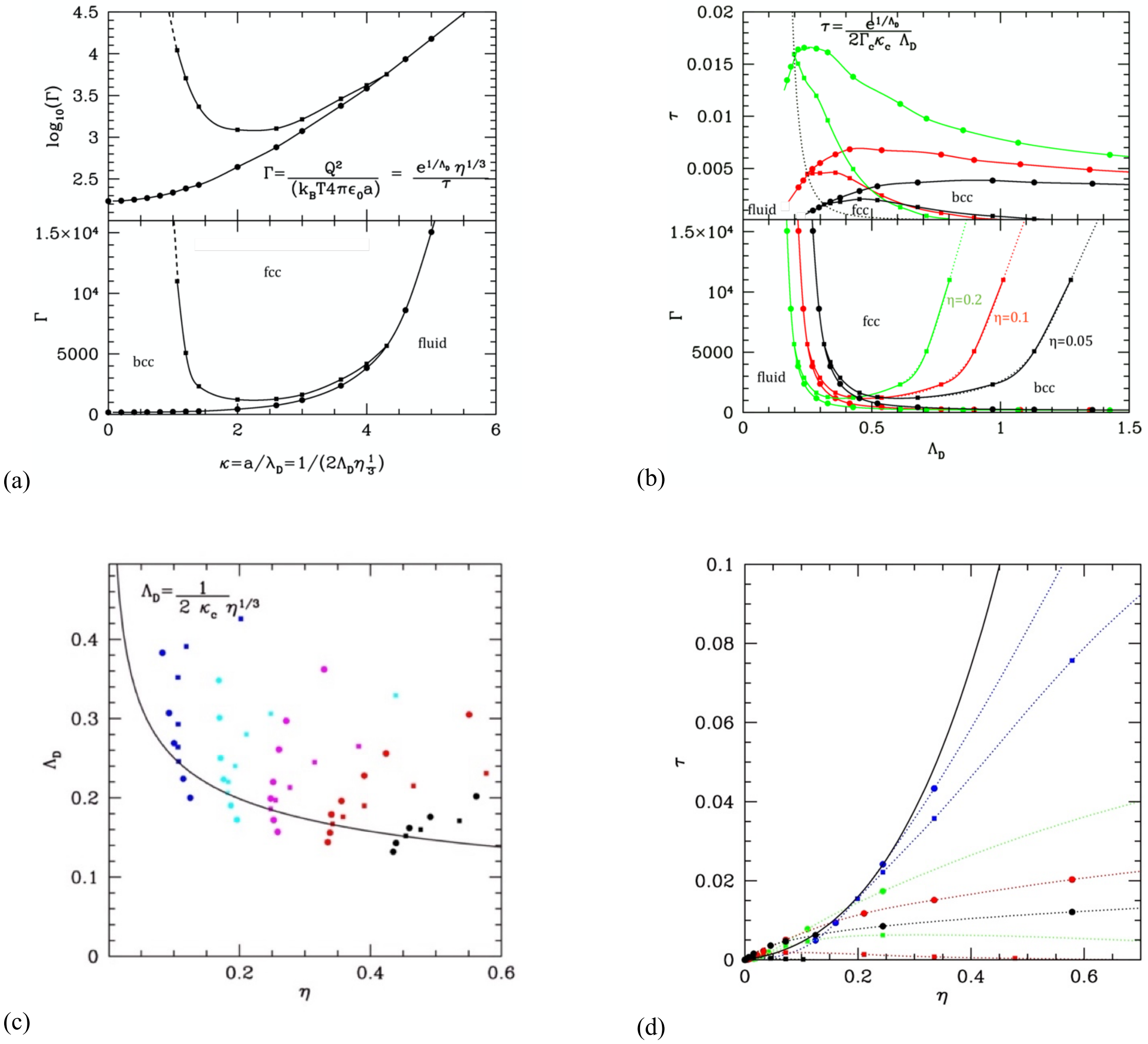

Supplementary Figure S.2 Phase diagram of the PY potential. (a) Phase diagram in the standard Γ-κ form, data taken in numerical form from[1]. The line with filled dots is the fluid-solid transition line, the one with filled squares is the fcc-bcc separation line (fcc phase above, bcc below). For values of κ larger than k_c and Γ larger than Γ_c the bcc phase is no more present. This triple point is located by the authors at k_c=4.28 and Γ_c=5.6x10^3. (b) report the phase diagram with the parameters of the HSY potential, i.e. the reduced Debye length and temperature, using the curves of (a) and the density dependent scaling laws of table S.1. The phase diagram, therefore, depends also on the reduced density η, and is reported for three values of it η (reported). The triple point in the τ-Λ_D plane lye on a curve (dotted line), whose analytical form is reported. On the left of the as the temperature decrease, one has the fluid→fcc transition; on the right of the line, the transition passes though the bcc phase fluid→bcc→fcc; as Λ_D increases the bcc phase region extends and the fcc phase tends to disappear. (c) The Λ_D(η) phase diagram at give temperatures (blue τ=0.005, cyan τ=0.013 magenta, τ=0.025, red τ=0.05, black τ=0.1. The critical line is reported in black. Below that line, only the fluid-fcc transition is present. (d) The τ- η phase diagram for different values of Λ_D (blue 0.2, green 0.4, red 0.6, black 1.0). The critical line is reported as black solid. As it can be seen for large Debye length, the fcc phase flattens to the horizontal axis and disappear. At variance with HSY, the transition at η~0.5 to the fcc phase is absent here, due to absence of the repulsive core.

Figure S.2 reports the results of the seminal work on the point-like Yukawa[1], shown in panel (a) using the numerical data taken from the paper. The system is conventionally represented in terms of the conventional dimensionless parameter Γ, the density reduced inverse temperature, and κ, the inverse density reduced Debye length. At small κ the system has a fluid-bcc-fcc transition as the temperature increase, up to a triple point value of κ over which the bcc phase disappear and the system displays the fluid-fcc transition; on the other way round, at infinite Debye Length (κ=0), i.e. in the pure coulomb limit, the fcc phase disappear. Using the density dependent units for temperature and length is customary in the PY due to the absence of a reference length σ. However, this can be reintroduced into the system using the relationships in Table S.1 (last row) to better compare with the HSY density dependent phase diagrams. Panel (b) reports the phase diagrams at three different densities, as a function of the temperature $\tau=k_BT/\varepsilon$ and of Γ. The fcc is the only solid phase present at small values of Λ_D (at the left of the triple point line, dotted), while it tend to disappear at large values of Λ_D. Finally, panels (c) and (d) reports the Λ_D-η and τ- η phase diagrams to compare with the corresponding ones of IPL and HSY. Overall at the reported densities they are quite similar to those of HSY, except for the fact that the vertical bending of lines due to the presence of the hard core is absent. In this respect, they behave more similarly to IPL, displaying softness. However, at variance with IPL, the fluid-fcc phase separation in this case is never visible for any value of the range, since PY does not tend to HS for small ranges.

S.2 Properties of the α-2α attractive potential

The α-2α potential is often used as an extension of Lennard Jones with variable range. LJ can be re-obtained with α=6

$$u^{\alpha}(r) = 4\varepsilon\left[\left(\frac{\sigma}{r}\right)^{2\alpha} - \left(\frac{\sigma}{r}\right)^{\alpha}\right] \tag{S.3}$$

vanishes in $r = \sigma$ and has a minimum where its derivative vanishes

$$\frac{d}{dr}\,u^{\alpha}(r) = -\frac{4\varepsilon}{\sigma}\left[2\alpha\left(\frac{\sigma}{r}\right)^{2\alpha+1} - \alpha\left(\frac{\sigma}{r}\right)^{\alpha+1}\right] \tag{S.4}$$

i.e. in $r = \sigma 2^{1/\alpha}$ $u^{\alpha}(r) = \varepsilon$. To evaluate the range we consider the distance of the flex point, i.e. where the second derivative vanishes

$$\frac{d^2}{dr^2}\,u^{\alpha}(r) = \frac{4\varepsilon}{\sigma^2}\left[2\alpha(2\alpha+1)\left(\frac{\sigma}{r}\right)^{2\alpha+2} - \alpha(\alpha+1)\left(\frac{\sigma}{r}\right)^{\alpha+2}\right] \tag{S.4}$$

i.e. $\Lambda = (2(2\alpha+1)/(\alpha+1))^{1/\alpha} - 1$, which can be approximated with $\Lambda \sim \ln(4)/\alpha$, for large α. However, at the flex point the potential assumes approximately the value -0.7ε, i.e. very near to the minimum of the well. In order to compare with the HAY range one should evaluate the distance at which the potential assume the value -0.5 ε (halving range). This is

$$\Lambda_{1/2} = \left[\frac{2}{1 - 1/\sqrt{2}}\right]^{1/\alpha} - 1 \tag{S.5}$$

and is reported in Figure S.3 (red) together with the flex point defined range (green). The ratio between the two ranges is approximately 3/2. We also observe that the flex point range is twice the distance between the wall and the minimum ($(r_0 - \sigma)/\sigma \sim \ln(2)/\alpha$) and the halving range about three times by the same quantity. A better correspondence with the HAY range could be obtained considering that at the range distance the HAY potential is decreased by a factor $1/(e(1+\lambda/\sigma))$, rather than halved. Therefore the corresponding range could be obtained increasing the halving range by a factor ~1/ln(2) at small ranges, i.e. overall $\Lambda_{\to HAY} \sim 3/\alpha$. For $\alpha = 6$ (standard LJ) one gets Λ =0.24, $\Lambda_{1/2}$ =0.37, $\Lambda_{\to HAY} \sim 0.5$.

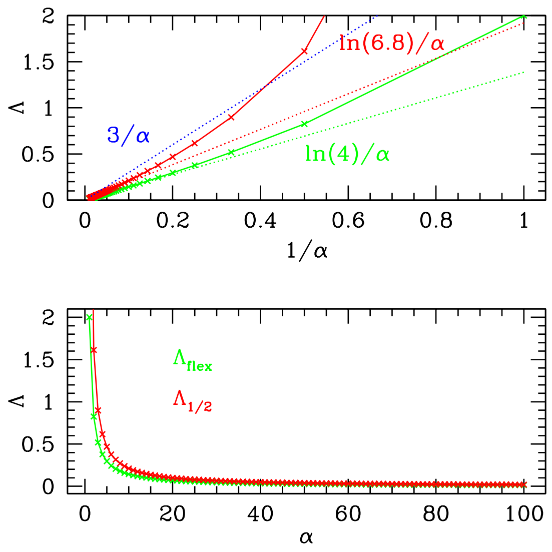

Supplementary Figure S.3 Range of the α-2α potential evaluated as the distance from the repulsive wall and the flex point, as a function of α and 1/α. The range evaluated from the position of the flex point is reported in green, the halving range in red, both measured with respect to the repulsive wall. In the upper plot they are reported as a function of the inverse α, and compared with their approximation for large α (dotted lines). Finally, the range that better corresponds to the Debye length in HSY, approximately 3/α, is reported in blue, dotted line.

S.3 Summary parametric phase diagram of purely attractive/repulsive potentials

Fig S.4 reports an expanded version of the parametric phase diagram (Fig 3 in the main text),as a function of different variables.

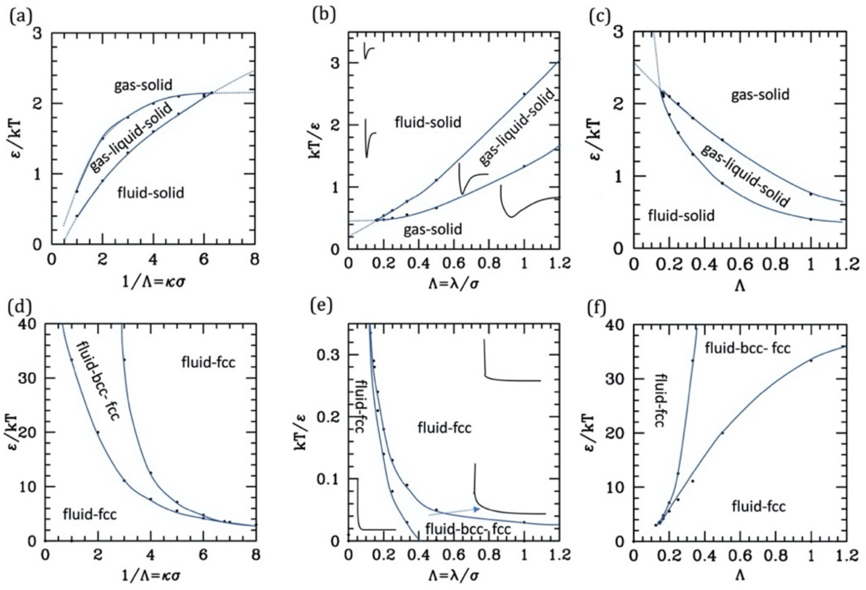

Fig S.4. Phase diagrams of the attractive (a,b,c) and repulsive (d,e,f) potentials, in the inverse range-strength (a,d) plane, and in the range-reduced temperature plane (b,e) and in the range-strength plane (c,f). Dots are numerical data extracted from ref[79,85]. Blue lines are eye guiding and the black lines report a qualitative representation of the corresponding potentials.

S.4 A generalized view of the SALR

Generally, the SALR potentials are represented as an additive combination of repulsive and attractive parts, the repulsive one represented with a point-like Yukawa (see main text, Table 2). This however modifies the well depth of the attractive part which is not decoupled by the repulsive one. A different possibility is to use a function switching attraction to repulsion at the distance corresponding approximately to the location of the barrier, i.e. $\sigma(1+\alpha\Lambda_0)$ with $\alpha\sim2$ so that no new parameters are introduced. The two possibilities are illustrated in Supplementary Table S.2. Here we use a Morse function in place of the generalized LJ or of the attractive Yukawa, since its parameters are more directly related to range and barriers. The main advantage in using the switch function in place of the sum is that the well remains unchanged, if the switching parameters are linked to the attractive range as reported in the table (i.e. $r_{sw}/\sigma = (1+\alpha\Lambda_0)/\beta\Lambda_0$ with $(\alpha\sim2, \beta\sim0.5)$). In the sum the attractive well is moved upward of a quantity that in first approximation is A (the repulsive strength), but if ranges are large can be very different. The barrier heigh also depends in a complex way from A and the ranges, but in first approximation it is A-0.3ε. Samples of potentials obtained with different parameters combinations are reported in the Table S.2 representative of the main classes of colloids also dicussed in the main text. We observe that due to the complex dependence of the barrier on the ranges ratio (an approximate version of this dependence is reported in the table) as the ratio Λ_0/Λ_1 increases, the barrier decreases, and larger values of a are needed to maintain it.

Supplementary Figure S.5 is an attempt to interpret the SALR behavior already highlighted in the main text, here given with some more detail. We regard the different kinds of SALRs as the superposition of the phase diagrams of the corresponding attractive and repulsive parts. Therefore we put in the first row and first column first of all the phase diagrams of the repulsive and attractive potentials at different ranges.

In the crossing cells of the matrix we report the superposition. The repulsive tail brings a new transition line (reported in red) homologous to the fluid-solid transition in purely repulsive potentials, superimposed to the dispersed-aggregated-solid (homologous of gas-liquid-solid) like diagram for purely attractive (in green). However, in making the superposition one must remember that the repulsive tail "starts" at the level of the barrier (located approximately at $\sigma+3\lambda_0$). Therefore in order to match the scale of $\eta\sim\rho\sigma^3$, the transition lines must be moved on the left by a factor $1/(1+3\Lambda_0)^3$, which brings the red line crossing the binodal line coming from the attractive part in different points depending on the attractive range. The low temperature part of this transition line, identified with the percolation line, separates the clustered with the cluster-percolate phase in the region corresponding to the fluid phase due to the attractive well. This picture is valid until the temperature remains lower than the barrier between attraction and repulsion. Conversely, at very high temperatures the system is expected to be dominated by the repulsive core at σ, therefore the percolation line must merge into the usual disperse-solid region. In the intermediate-but-supercritical region, a transition behavior, which may cause the percolation line effectively bend toward the right.

	**Attractive**	**Repulsive**	**Sum**	**Switch**
	$u^{att}_{\varepsilon,\Lambda_0}\left(\frac{r}{\sigma}\right) = \varepsilon\left[\left[e^{-\frac{r-(\sigma+\lambda_0 \ln(2))}{\lambda_0}} - 1\right]^2 - 1\right]$	$u^{rep}_{A,\Lambda_1}\left(\frac{r}{\sigma}\right) = A\frac{e^{-(\frac{r}{\sigma}-1)/\Lambda_1}}{r/\sigma}$	$u_{\varepsilon,a,,\Lambda_0,\Lambda_a} = \varepsilon\left[u^{att}_{\varepsilon,\Lambda_0} + a\, u^{rep}_{A,\Lambda_1}\right]$ $a = A/\,\varepsilon$	$u_{\varepsilon,a,,\Lambda_0,\Lambda_1}$ $= \varepsilon\left[u^{att}_{\varepsilon,\Lambda_0} + f^{sw} a\, u^{rep}_{A,\Lambda_1}\right]$ $f^{sw}(x) = \frac{e^x}{e^x + e^{-x}}$ $x = \frac{\frac{r}{\sigma} - (1+\alpha\Lambda_0)}{\beta\Lambda_0}$ $(\alpha\sim 2, \beta\sim 0.5)$
Repulsive core	$\frac{r}{\sigma} = 1, \quad r = \sigma$		$r \approx \sigma\left(1 + \frac{a\Lambda_0}{2}\right)$ a<1, $\Lambda_0 \ll 1$	$\frac{r}{\sigma} = 1, \quad r = \sigma$
Well depth	$r_0 = (\sigma + \lambda_0 \ln(2))$ $u_0 = \varepsilon$		$r \approx \sigma\left(1 + \frac{a\Lambda_0^2}{4}\right)$ a<1, $\Lambda_0 \ll 1$ $u_0 = \varepsilon - A$	$r_0 = (\sigma + \lambda_0 \ln(2))$ $u_0 = \varepsilon$
Barrier			$r_B \sim (\sigma + 3\lambda_0)$ $u_0 \sim -0.2\varepsilon + A\frac{e^{-\frac{3\Lambda_0}{\Lambda_1}}}{1+3\Lambda_0}$	$r_B \sim (\sigma + 3\lambda_0)$ $u_0 \sim -0.2\varepsilon + A\frac{e^{-\frac{3\Lambda_0}{\Lambda_1}}}{1+3\Lambda_0}$
Short range attraction Long range strong repulsion	Λ0=0.1	Λ1=1.5 A=0.9	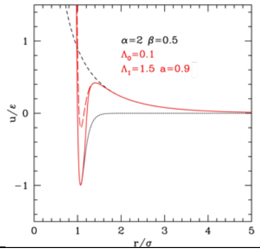	
average range attraction <br>average repulsion	Λ0=0.2	Λ1=1.3 <br>A=0.9	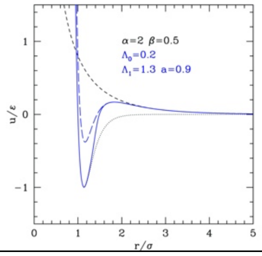	
Long range attraction	Λ0=0.4	Λ1=1.3 <br>A=1.1	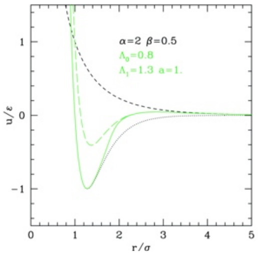	

Supplementary Table S.2 properties of SALRs potentials of different classes in sum or switch combination. Barrier are evaluated considering that the switch function definitely switches to 1 at the distance $\sigma + \alpha\lambda_0 + 2\beta\lambda_0 \sim \sigma + 3\lambda_0$ with the chosen values of α and β $(0.2 \sim 3e^{-3})$. In the plots, the solid colored lines are the competing potential obtained with the switch function, while the colored dashed are obtained with the simple sum. Values of parameters reported.

The plots corresponding to the parameters typically describing colloids are highlighted in yellow. The four phases most commonly identified in the literature studies (disperse, percolate, clusters and cluster percolated) are separated by the percolation line (in red in the plots) and the binodal line (in green). However, additional features can appear. For instance for colloids with extremely small attractive range a “two phase” (“liquid-liquid”) region can appear within the binodal region; the long range of the repulsive tail enlarges the coexistence crystalline region at low temperature possibly introducing different symmetries in the clusters or percolates. We

observe that the liquid-liquid and the repulsive crystalline coexistence region may superimpose, appearing both a low temperatures and intermediate densities with very short range attractive and long range repulsive potentials, giving rise to a rich variety of possible phases. Some of these phases may appear as metastable transients and are seen to influence the mechanism of gelation[2].

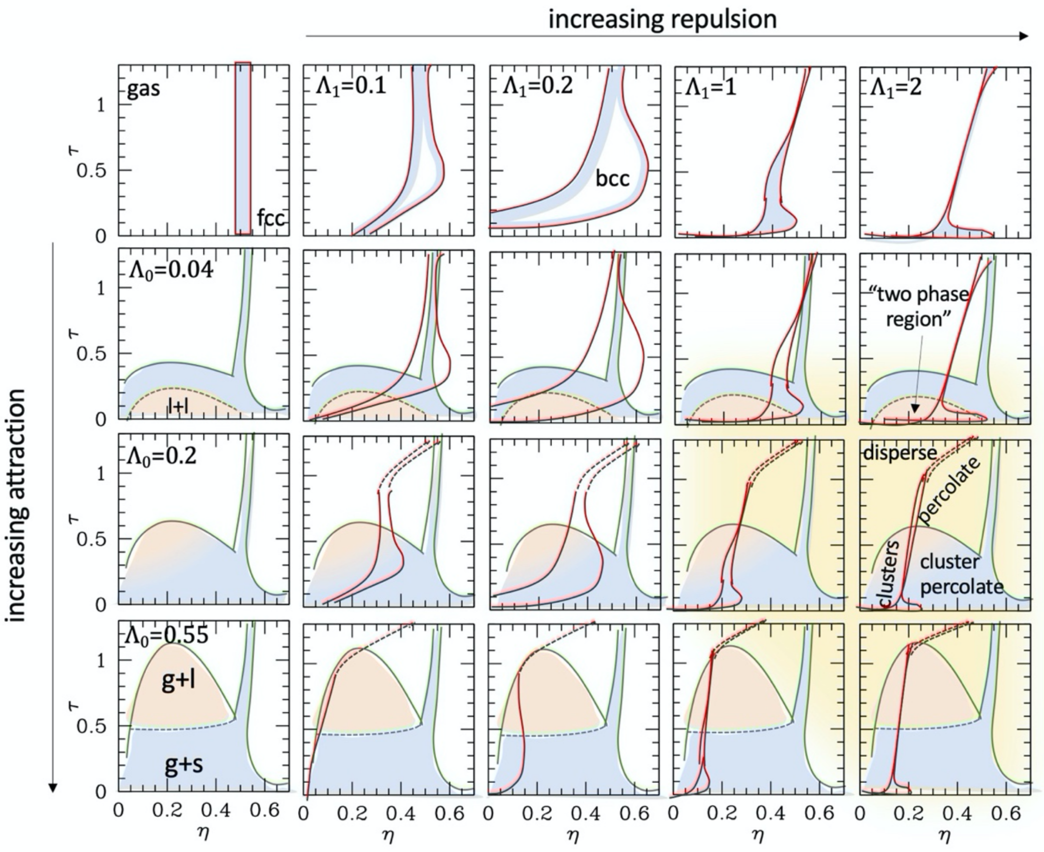

Supplementary Figure S.5 Combination of the repulsive and attractive potentials at different strength and ranges to aid the interpretation of SALRs phase diagram. On the first row, the purely repulsive potentials are reported as s function of the range (inverse range values are given, the range increases from left to right). On the first column attractive potentials of increasing range (inverse range is reported, increasing from top to bottom). In the matrix, the combinations are reported. In each case the repulsive part is shifted at the location of the barrier, considered as the effective starting point of the repulsive tail. Highlighted in yellow are the plots corresponding to typical colloids.

Conversely, when one of the two components is very short ranged/weak the corresponding phase diagrams of the other "pure" potential is returned. If the attractive range becomes very weak, the binodal line lowers to extremely low temperature and only the percolation line remains to separate the disperse from the percolate (or gel) phase; conversely when the repulsive range becomes weak the percolation line tend to disappear.

Supplementary references

[1] Triple point of Yukawa systems S. Hamaguchi, R. T. Farouki, and D. H. E. Dubin , Phys Rev E, 56 4671, 1997
[2] , Das et al (*Soft Matter,* 2018, 14, 92)
